# Defining the molecular basis of interaction between R3 receptor-type protein tyrosine phosphatases and VE-cadherin

**DOI:** 10.1371/journal.pone.0184574

**Published:** 2017-09-19

**Authors:** Olga Dorofejeva, Alastair J. Barr

**Affiliations:** Department of Biomedical Science, Faculty of Science & Technology, University of Westminster, London, United Kingdom; Thomas Jefferson University, UNITED STATES

## Abstract

Receptor-type protein tyrosine phosphatases (RPTPs) of the R3 subgroup play key roles in the immune, vascular and nervous systems. They are characterised by a large ectodomain comprising multiple FNIII-like repeats, a transmembrane domain, and a single intracellular phosphatase domain. The functional role of the extracellular region has not been clearly defined and potential roles in ligand interaction, dimerization, and regulation of cell-cell contacts have been reported. Here bimolecular fluorescence complementation (BiFC) in live cells was used to examine the molecular basis for the interaction of VE-PTP with VE-cadherin, two proteins involved in endothelial cell contact and maintenance of vascular integrity. The potential of other R3-PTPs to interact with VE-cadherin was also explored using this method. Quantitative BiFC analysis, using a VE-PTP construct expressing only the ectodomain and transmembrane domain, revealed a specific interaction with VE-cadherin, when compared with controls. Controls were sialophorin, an unrelated membrane protein with a large ectodomain, and a membrane anchored C-terminal Venus-YFP fragment, lacking both ectodomain and transmembrane domains. Truncation of the first 16 FNIII-like repeats from the ectodomain of VE-PTP indicated that removal of this region is not sufficient to disrupt the interaction with VE-cadherin, although it occurs predominantly in an intracellular location. A construct with a deletion of only the 17th domain of VE-PTP was, in contrast to previous studies, still able to interact with VE-cadherin, although this also was predominantly intracellular. Other members of the R3-PTP family (DEP-1, GLEPP1 and SAP-1) also exhibited the potential to interact with VE-cadherin. The direct interaction of DEP-1 with VE-cadherin is likely to be of physiological relevance since both proteins are expressed in endothelial cells. Together the data presented in the study suggest a role for both the ectodomain and transmembrane domain of R3-PTPs in interaction with VE-cadherin.

## Introduction

It is well established that protein tyrosine phosphorylation, an event brought about by tyrosine kinases and reversed by phosphatases, plays a critical role in many physiological processes [1, 2]. The large family of human protein tyrosine phosphatases (PTPs) catalyse dephosphorylation and their activity and specificity is tightly regulated by a range of mechanisms [3–5]. PTPs have been divided into transmembrane receptor-type PTPs (RPTPs, subgroups R1-R8) and intracellular non-transmembrane PTPs (subgroups NT1-NT9), based on sequence similarity and the presence of similar structural and functional domains. The receptor-type PTPs have highly-variable ectodomains, a single transmembrane spanning region, and an intracellular region which may contain either one or two phosphatase domains [6]. Although much is known about the structure, function and substrate specificity of the phosphatase domain [7], the function of the extracellular region within many RPTPs is only beginning to be uncovered. Roles have been described for this region in binding a diverse range of ligands either *in trans* or *in cis*, mediating cell-cell contact, oligomerization, and steric exclusion of the phosphatase from an immunological synapse [8–14].

The R3 subgroup of RPTPs includes density-enhanced phosphatase 1 (DEP-1), vascular endothelial protein tyrosine phosphatase (VE-PTP), stomach cancer-associated protein tyrosine phosphatase 1 (SAP-1), glomerular epithelial protein 1 (GLEPP1), and receptor-type protein tyrosine phosphatase Q (PTPRQ). They share a similar structure composed of a heavily glycosylated ectodomain containing a variable number of fibronectin III (FNIII)-like repeats, a transmembrane spanning domain, and a single intracellular phosphatase domain. The number of FNIII repeats ranges from 8 in GLEPP1, which also has a truncated isoform lacking the ectodomain, to 17 in VE-PTP and 18 in PTPRQ. The prototypical FNIII domain is found in the extracellular matrix protein fibronectin, and cells interact with this molecule via integrin α5β1 and αVβ3 binding to the RGD (Arg-Gly-Asp) sequence in exposed loop of FNIII-10 and the synergy site in FNIII-9 [15]; however, the role of the related FNIII repeats in R3-PTPs has yet to be clearly defined. For DEP-1, it has been reported that the ligands thrombospondin and syndecan-2 interact with the ectodomain and modulate phosphatase activity [16, 17]. Antibodies directed to this region either increase or decrease phosphatase activity depending on the system studied [18, 19]. Furthermore, genetic polymorphisms in the DEP-1 ectodomain have been characterised, which do not have an effect on cell surface expression, but are associated with a decrease in phosphatase activity and a protective effect from adverse drug reactions to heparin [20]. Other studies with SAP-1 have documented ectodomain-dependent dimerization leading to a decrease in phosphatase activity [21] while an alternative model suggests that the ectodomain of R3-PTPs, in particular DEP-1, plays a role in excluding these molecules from signalling complexes [12]. Collectively, these studies highlight that the ectodomain plays an important role in regulating activity although the molecular basis of this is unclear.

**Fig 1:**
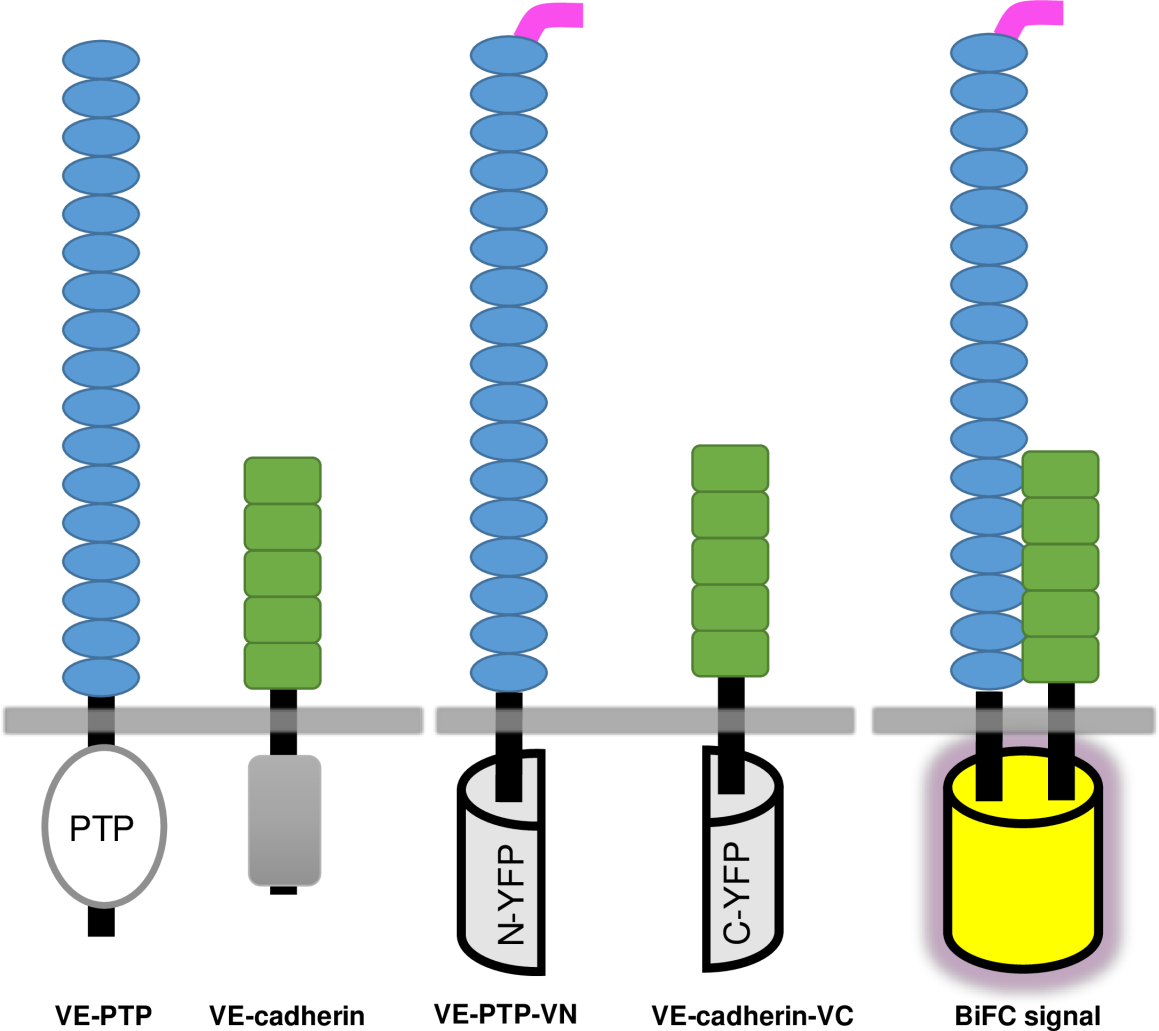
Representation of VE-PTP and VE-cadherin constructs used, and the BiFC principle. VE-PTP consists of multiple extracellular fibronectin III-like repeats (blue), a transmembrane domain and an intracellular protein tyrosine phosphatase domain (PTP). VE-cadherin consists of 5 extracellular cadherin domains (green), a transmembrane sequence and an intracellular cadherin cytoplasmic region (grey). The constructs VE-PTP-VN and VE-cadherin-VC have a modified N- or C-terminal sequence of the Venus protein (a yellow fluorescent protein (YFP) variant; represented by a half cylinder) inserted five residues after the transmembrane domain. A myc-tag (pink) has been inserted after the signal peptide of VE-PTP to facilitate analysis of expression. When brought into close proximity by a pair of interacting proteins, the fragments assemble to yield a fluorescent protein (BiFC signal).

R3-PTPs play many important physiological roles (reviewed in [1, 22]). VE-PTP has been shown in both *in vitro* and *in vivo* studies to maintain endothelial cell contact integrity via an interaction with the cell adhesion molecule VE-cadherin [23–25], and modulating this interaction has potential as a therapeutic strategy in inflammation and oedema [26]. Co-immunoprecipitation studies have determined that these two proteins interact through their ectodomains, specifically the 17^th^ FNIII domain of VE-PTP and 5^th^ cadherin domain of VE-cadherin [27]. In our study the VE-PTP/VE-cadherin pair was used as an exemplar to investigate R3-PTP ectodomain protein-protein interactions using bimolecular fluorescence (BiFC). This technique, based on the reconstitution of a fluorescent protein from non-fluorescent fragments (Fig 1), has been widely used to study protein-protein interactions and incorporates the advantages that it can be used in living cells (avoiding the need for harsh detergents that may disrupt interactions or give artefactual results), and provides information on the sub-cellular site of a protein interaction event [28]. The technique is not without shortcomings, however, the major drawback being that non-fluorescent fragments can reassemble in the absence of a *bona fide* protein-protein interaction. Although more recent versions of the system have aimed to address this issue [29], it is recognised that appropriate controls are essential [30]. Using the BiFC technique we demonstrate specific VE-PTP interactions with VE-cadherin via the ectodomain and transmembrane domain, investigate the region of the ectodomain involved in interactions, and show that other R3-RPTPs have the potential to interact with VE-cadherin. The study also highlights the challenge of using the BiFC technique with transmembrane proteins and particularly the crucial use of appropriate internal controls and quantitation.

## Materials and methods

### Antibodies

Mouse monoclonal anti-myc antibody (4A6) was purchased from Millipore. Anti-HA antibody (12CA5), anti-VE-cadherin antibody (BV9) and anti β-actin antibody were obtained from Abcam. Goat anti-mouse IgG-HRP linked secondary antibody was obtained from Sigma.

### DNA constructs

Human cDNAs for VE-PTP (BC096364.3), GLEPP1 (BC126203), SAP-1 (BC111716), sialophorin (BC012350) and VE-cadherin (BC096364.3) were purchased from SourceBioscience. A synthetic sequence was inserted into the BC096364.3 clone to generate a full-length gene sequence that corresponds to P33151-1. A construct encoding human DEP-1 was kindly provided by A. Weiss (UCSF, CA). The pBiFC-VN155 (1–154, I152L, #27097) and pBiFC-VC155 (155–238, A206K, #22011) vectors encoding N- and C-terminal fragments of the Venus yellow fluorescent protein (YFP) respectively were from Addgene [29].

A myc-tag (EQKLISEEDL) in pBiFC-VN155 or HA-tag (YPYDVPDYA) in pBiFC-VC155 was inserted after the signal peptide for each of the constructs by cloning a synthetic DNA sequence (S1 Table) purchased from Genscript into either the *Apa*I*/Xho*I or *Apa*I*/Eco*RI restriction enzyme sites of the pBiFC vectors. The ectodomain and transmembrane coding sequences for the above proteins were amplified by PCR using Platinum Pfx DNA Polymerase (Invitrogen), and subsequently inserted into the *Xho*I site or *Eco*RI*/Xho*I sites of the vectors. Oligonucleotide primer sequences are listed in Table 1. The VE-cadherin construct was generated without an epitope tag and was cloned directly into the *Apa*I*/Kpn*I of pBiFC-VC155. Construct sequences were confirmed by Sanger sequencing (GATC Biotech).

**Table 1:** Oligonucleotide primer sequences.

Construct name	Primer: Sequence (5’->3’)	Restriction sites
VE-PTP-VN or VC	For: AGTATCTCGAGGTAGATGTAACTTCACCCTGGCG	*Xho*I */ Xho*I
VE-PTP-VN or VC	Rev: TAGCACCTCGAGAGCTCACTTTCTGTCTGCAGATC	*Xho*I */ Xho*I
VE-PTP-VN (Δ17FN)	For: GATCCCCGGGCTAGGTGGAAAATGCGATCC	*Xma*I
VE-PTP-VN (Δ17FN)	Rev: GATCCCCGGGCACACGAATGTGTGGGGGTG	*Xma*I
VE-PTP-VN (5-17FN)	For: GATCCCCGGGAAAGTTGCAAACCTGGAGGC	*Xma*I
VE-PTP-VN (5-17FN)	Rev: GATCCCCGGGAGACAGGTCCTCCTCTGAGATC	*Xma*I
VE-PTP-VN (10-17FN)	For: GATCCCCGGGCCAGAGCCTGTTAAGGATCTAACA	*Xma*I
VE-PTP-VN (10-17FN)	Rev: GATCCCCGGGAGACAGGTCCTCCTCTGAGATC	*Xma*I
VE-PTP-VN (17FN)	For: GATCCCCGGGAATGAAAAGGATGTGCTAATTAGCA	*Xma*I
VE-PTP-VN (17FN)	Rev: GATCCCCGGGAGACAGGTCCTCCTCTGAGATC	*Xma*I
DEP-1-VN & VC	For: CTGATCTCAGAGGAGGACCTGTCTCGAGGTACCCCTAGTCCAATTCCTGA	*Xho*I */ Xho*I
DEP-1-VN & VC	Rev: CTCCGGTACCTCGAGATTTCCTCTTCTTTCTCCAGAAGA	*Xho*I */ Xho*I
GLEPP1-VN & VC	For: CTGATCTCAGAGGAGGACCTGTCTCGAGGTACTGTCCAAGATGATAATAACATCG	*Xho*I */ Xho*I
GLEPP1-VN & VC	Rev: CTCCGGTACCTCGAGAATGCTTTTTCCTAAGAATAATGAGG	*Xho*I */ Xho*I
SAP-1-VN & VC	For: CTGATCTCAGAGGAGGACCTGTCTCGAGGTAACCCAGGGAGGAACCTG	*Xho*I */ Xho*I
SAP-1-VN & VC	Rev: CTCCGGTACCTCGAGAATTCCTCCTCTTCAGGAAGAAAA	*Xho*I */ Xho*I
VE-cadherin-VN & VC	For: GATCGGGCCCACCATGCAGAGGCTCATGATGCTC	*Apa*I */ Kpn*I
VE-cadherin-VN & VC	Rev: GATCGGTACCCCGGAGCCGCCGCCGCAGGAAG	*Apa*I */ Kpn*I
SPN-VN & VC	For: GATCCGAATTCGGGCAGTGCAGACACCCACCTC	*Eco*RI */ Xho*I
SPN-VN & VC	Rev: GATCACCTCGAGACTGCCGCCGGCGCCACAGCA	*Eco*RI */ Xho*I
myr-VN & VC	For: CACCATGGGCTGTGGCTGCAGCTCACACCCGGAAGATGACTGGGAGCAGAAGCTGATCTCAGAGGAGGACCTGCG	*Apa*I */ Eco*RI
myr-VN & VC	Rev: AATTCGCAGGTCCTCCTCTGAGATCAGCTTCTGCTCCCAGTCATCTTCCGGGTGTGAGCTGCAGCCACAGCCCATGGTGGGCC	*Apa*I */ Eco*RI

Constructs consisting of ectodomain and transmembrane domain sequences, and truncation and deletion constructs, were generated by PCR using the indicated primer pairs below as indicated in the materials and methods. PCR products were inserted into pBiFC-VN155 with the corresponding signal peptide and myc-tag, pBiFC-VC155 with the corresponding signal peptide and HA-tag or both vectors using the restriction enzymes indicated (underlined). The Lck myrsitoylation sequence is shown double-underlined.

Deletion and truncation mutants of VE-PTP were generated by PCR [31]. Primers (Table 1) contained a non-complimentary 5’ *Xma*I restriction enzyme site. Following PCR amplification using the VE-PTP-VN construct as a template the product was digested with *Dpn*I*/Xma*I restriction enzymes and ligated to create expression vectors for the deletion or truncation mutants indicated. Membrane anchored pBiFC-VN155 or pBiFC-VC155 constructs containing the Lck myristoylation sequence followed by a myc-tag were generated by cloning a synthetic adaptor DNA sequence (Table 1) into the *Apa*I*/Eco*RI of the vector.

### Cell culture

Human Embryonic Kidney 293T (HEK293T) cells (ATCC code CRL-11268) were maintained in Dulbecco's Modified Eagle's Medium (DMEM, GIBCO), containing GlutaMAX, high glucose, sodium pyruvate and supplemented with 10% (v/v) fetal bovine serum at 37°C and 5% CO_2_ in a humidified incubator. Cells were trypsinized at 80% confluence and seeded 24 hours prior to transfection on 6-well (3 x 10^5^ cells / well) plates.

### Transfection and confocal microscopy

Transient transfections were performed using Lipofectamine LTX Plus (Invitrogen) in 500 μl Optimem according to the manufacturer’s instructions with 0.5 μg of each construct. After 24 hours cells were stained with CellMask™ Deep Red plasma membrane stain (ThermoFisher Scientific) for 1 hour. The media was then removed, cells were rinsed with warm Hanks' Balanced Salt Solution (HBSS) and images acquired by sequential scanning using a Leica TCS SP2 confocal system with a 63× ceramic dipping objective. A 514 nm Argon ion laser was used to excite Venus YFP (Ex/Em 514/527 nm) with detection at 525–560 nm; and a 633 nm Helium Neon laser, intensity 35%, was used for the red stain (Ex/Em 649/666 nm) with detection at 660–685 nm, together with a dichroic beamsplitter DD458/514.

### Data analysis

For each combination of BiFC constructs analyzed, three images were obtained from three or more independent experiments. Fiji image analysis software (https://imagej.net/Fiji) was used to trace the border of each cell (typically 300 cells per experiment) and determine the average pixel intensity in both the yellow and red channels of the selected region. A background value from a region with no cells was subtracted. Values for both the yellow/red ratio for each region of interest and mean BiFC fluorescent intensity for each pairing were then calculated. Data was compared using IBM SPSS Statistics 22 software, using the Mann-Whitney U test. A value of P<0.05 was considered statistically significant for any set of data.

### SDS-PAGE and western blotting

Following transfection cells were washed with HBSS and lysed in either 200 μl RIPA buffer or 300 μl 2x Laemmli buffer (62.5 mM Tris-HCl, pH 6.8; 25% glycerol; 2% SDS, 0.01% bromophenol blue, and 5 mM DTT). The total cell lysate (20 μl) was heated to 95°C for 2 minutes and loaded onto a 4–20% Mini-Protean TGX precast SDS-PAGE gel (Bio-Rad) and subjected to electrophoresis at 110V for 60 minutes. Proteins were transferred to PVDF membranes (GE Healthcare) in transfer buffer (25 mM Tris, 192 mM glycine, 20% v/v methanol) for 2 hours at room temperature. Membranes were blocked with 3% w/v non-fat dry milk in Tris-buffered saline (TBS) and incubated with specific primary antibodies for 1 hour at room temperature. Following washing with TBS containing 0.05% Tween-20 (TBS-T) membranes were incubated with peroxidase-linked secondary antibody and developed with SuperSignal West Femto Maximum Sensitivity ECL Substrate (ThermoScientific) and visualized using an imaging system (UVP) with Labworks 4.1.

### Flow cytometry

Transiently transfected HEK293T cells were detached with Accutase, centrifuged for 5 mins at 400 g and resuspended in ice-cold FACS buffer (PBS supplemented with 0.2% BSA and 0.02% sodium azide). Cells (5 x 10^5^ in 50 μl) were incubated for 30 mins with anti myc-tag antibody (clone 4A6, Millipore) at a 1:100 dilution and then washed with 900 μl of FACS buffer and incubated with goat anti-mouse Alexa fluor-488 (Invitrogen) at 1:200 dilution for 30 mins on ice in the dark. An isotype control antibody and non-transfected cells were used as controls to validate antibody specificity. Cells were diluted in 450 μl FACS buffer and analysed on a BD Accuri C6 Plus flow cytometer. Cells were gated using forward scatter (FS) and side scatter (SS) to exclude dead cells and cellular debris.

## Results

### Co-expression of VE-PTP and VE-cadherin BiFC constructs results in fluorescence complementation

In the BiFC studies the Venus protein, a variant of YFP, and an improved BiFC system was used which incorporates mutants specifically engineered to reduce self-assembly, decrease background fluorescence, and increase signal to noise [29]. The constructs VE-PTP-VN and VE-cadherin-VC, shown diagrammatically, Fig 1, comprise residues 1–1647 of VE-PTP and 1–625 of VE-cadherin, with either the modified N- or C-terminal sequence of vYFP inserted five residues after the transmembrane domain and a flexible linker sequence, as used by Kodama & Hu [29]. This approach essentially replaced the intracellular region of these proteins with non-fluorescent fragments of vYFP, enabling investigation of protein-protein interactions that involve only the ectodomain and transmembrane domains.

**Fig 2:**
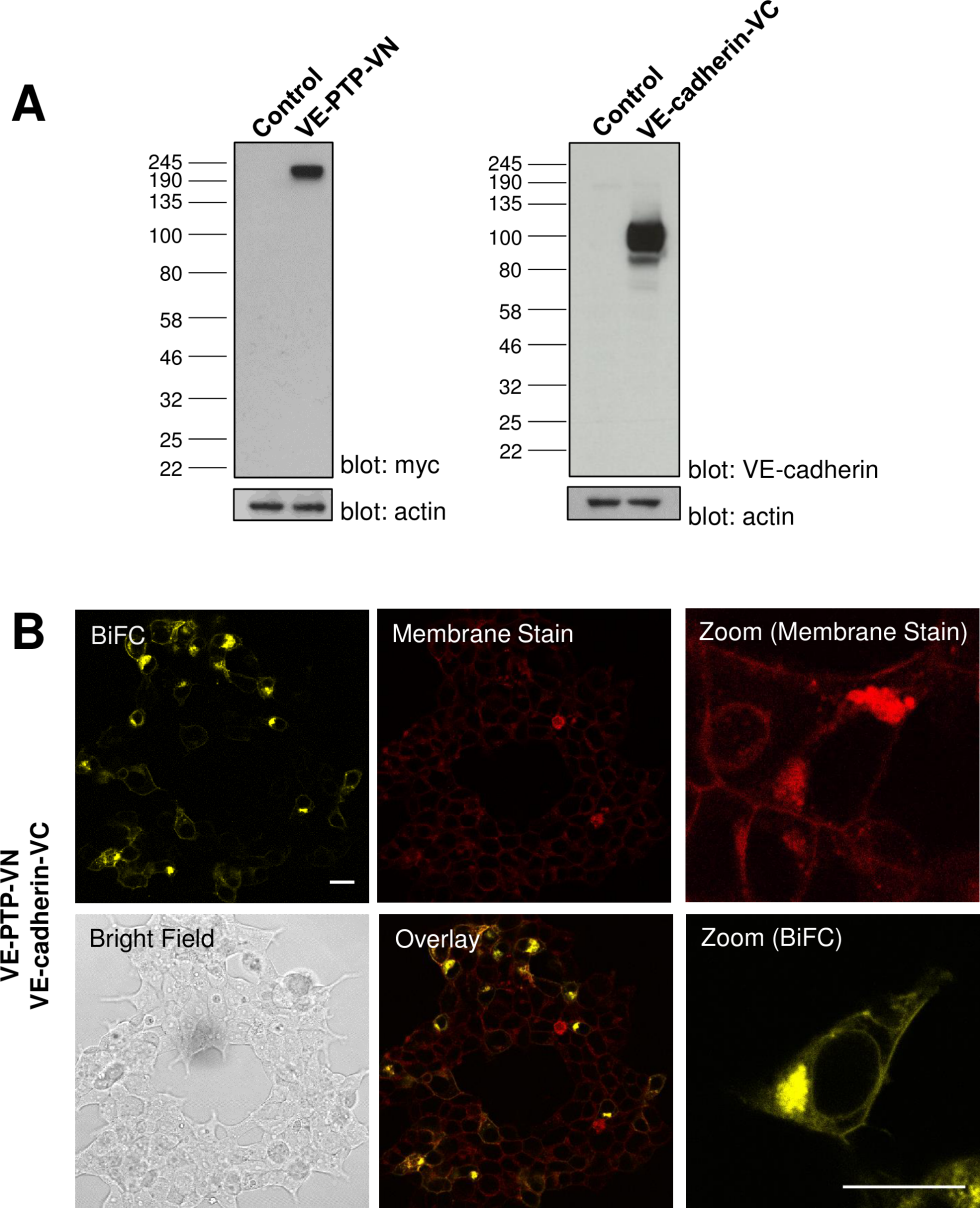
Co-expression of VE-PTP-VN and VE-cadherin-VC constructs yields a BiFC signal. **(A)** Western blotting of lysates from transfected HEK-293T cells confirmed the expression of both BiFC constructs at the expected molecular weight with β-actin was used as a loading control. **(B)** Constructs expressing ectodomain and transmembrane domains of VE-PTP and VE-cadherin as fusions with fragments of Venus YFP were transfected into HEK-293T cells. The constructs complement each other to yield a BiFC signal. Plasma membrane staining, brightfield and overlayed images are shown on additional panels together with a representative subfield at higher digital zoom showing detail at the single cell level. Data are representative of at least three independent experiments. Scale bar 20 μm.

Constructs were tested for expression by transiently transfecting plasmids into HEK-293T cells and lysing cells after 24 hours and subjecting the total cell lysate to SDS-PAGE and western blotting. Western blotting with an anti-myc antibody detected a protein with a molecular weight of approximately 210 kDa, corresponding closely to the predicted size for the VE-PTP-VN construct, which has a myc-tag inserted following the signal peptide sequence. The VE-cadherin antibody detected a protein of approximately 100 kDa, which is larger than the predicted size of 82 kDa. A similar difference has previously been documented in western blots of the full-length protein [32] and likely reflects glycosylation (Fig 2A). Co-expression of VE-PTP-VN and VE-cadherin-VC in HEK-293T cells resulted in a fluorescence complementation signal detectable by confocal microscopy which was stronger 24 hours post-transfection (Fig 2B), when compared with experiments conducted after 48 hours. Comparison of the sub-cellular localization of the Venus YFP signal with that of the CellMask DeepRed plasma membrane stain indicated that the fluorescence complementation signal was detected in the plasma membrane and intracellular structures, likely to be the rough endoplasmic reticulum (ER), of co-transfected cells. As anticipated, no detectable vYFP fluorescence was observed when plasmids were transfected individually (data not shown).

### Quantitation of the VE-PTP and VE-cadherin BiFC signal relative to control proteins

A key limitation of the BiFC technique is that the non-fluorescent protein fragments have the potential to associate with each other independent of an interaction between proteins fused to the fragments [33, 34]. This so-called “self-assembly” can lead to a high background signal in the absence of a *bona fide* protein-protein interaction. The BiFC technique has been extensively used to visualize protein-protein interactions involving membrane proteins; however, since the sub-cellular location of these proteins is restricted to the plane of the membrane it is likely that the background signal may be more significant than for cytosolic proteins [33]. Consequently it is essential to determine whether the BiFC fluorescence signal reflects the protein-protein interaction under study or a self-assembly background signal [30, 35]. This issue was addressed using several approaches: as above, an improved Venus-based BiFC system with designed mutations in the vYFP fragments to reduce self-assembly was used [29]; control experiments were performed in which the cognate interaction was examined in parallel with experiments using non-specific partner proteins, and rather than relying on qualitative analysis of results, the BiFC signal was quantitated relative to the control experiments.

**Fig 3:**
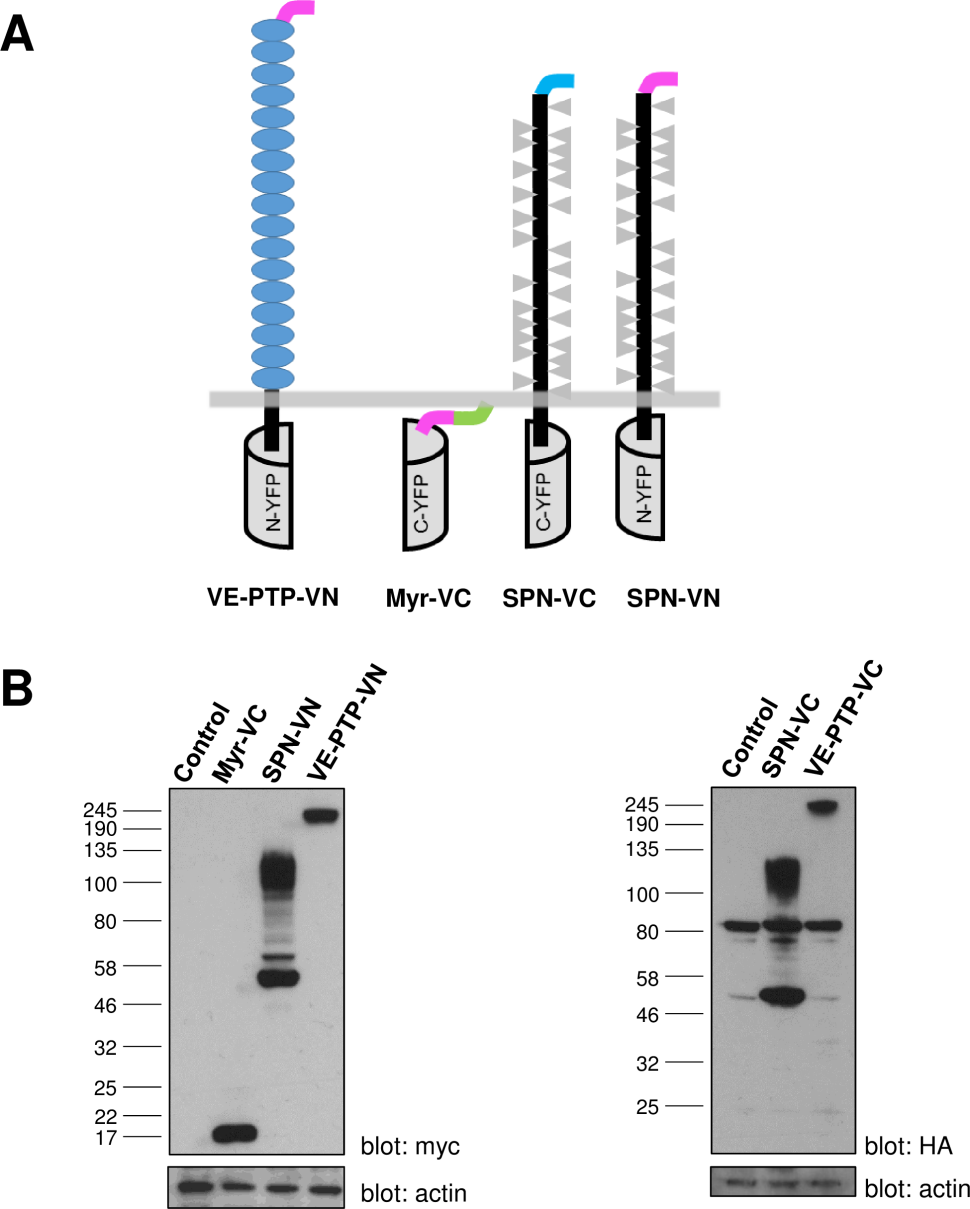
Representation of the control BiFC constructs and expression analysis. **(A)** The membrane-associated control (Myr-VC) lacks any extracellular and transmembrane regions, and has the Lck myristoylation sequence (green) preceeded by a myc tag (pink). Sialophorin (SPN) consists of a large highly glycosylated (grey triangles) ectodomain and single transmembrane spanning region. These constructs were fused to either the N- or C-terminal sequence of the Venus YFP protein and contained either an N-terminal HA-tag (blue) or myc-tag (pink). **(B)** Western blotting of cell lysates from transfected HEK-293T cells with either anti-myc or anti-HA antibodies confirmed the expression of the constructs at the expected molecular weight. Expression of VE-PTP-VC is shown for reference with β-actin used as a loading control.

As a control in the BiFC experiments, sialophorin (SPN) (also termed leukosialin and CD43) as a fusion with the N- or C-terminal vYFP sequence (designated SPN-VN and SPN-VC respectively) was used. The SPN protein is similar to R3-PTPs in that it has a large highly-glycosylated ectodomain and a single transmembrane spanning region; however, it has no homology to VE-PTP, or other R3-PTPs, and for these reasons has previously been used as a control in other studies of R3-PTP function [36]. Another control construct, entirely lacking any ectodomain and transmembrane regions, that was associated with the cell membrane via the Lck myristoylation sequence (designated myr-VC) was also used. The constructs are shown diagrammatically (Fig 3A) and western blotting of lysates from transiently transfected HEK-293T cells confirmed expression of myr-VC close to the predicted molecular weight (15 kDa). More than one band on the western blot was detected for both of the sialophorin constructs (SPN-VN and SPN-VC) with the predominant bands at approximately 50 kDa and 110 kDa. This is significantly larger than the predicted molecular weight (40 kDa), likely reflecting the extensive glycosylation of the molecule, as observed in other studies [37], and different glycosylation state as is commonly detected with membrane proteins in heterologous expression systems (Fig 3B). A non-specific band, present in un-transfected cells, was detected in HEK-293T when using the HA-tag antibody and this has been documented previously [38]. Control proteins were expressed at higher levels than test proteins.

**Fig 4:**
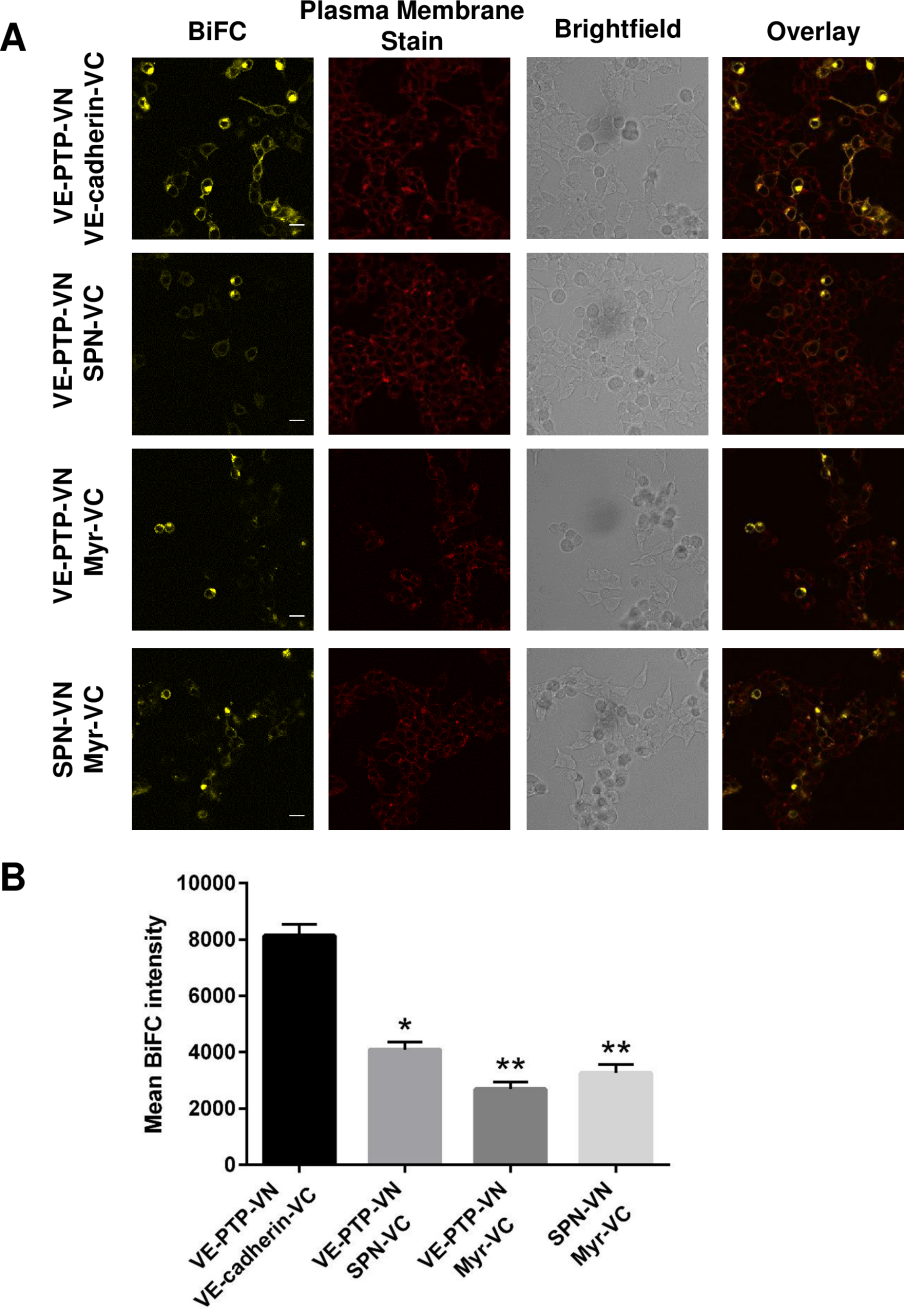
Comparison of BiFC signals from the VE-PTP and VE-cadherin with VE-PTP and control constructs. **(A)** Pairs of constructs were transfected into HEK-293T cells and the BiFC signal assessed. Plasma membrane staining, brightfield and overlayed images are shown on additional panels. Data are representative of at least three independent experiments; scale bar 20 μm. **(B)** Bar graphs represent the average BiFC fluorescence intensity from all cells in three images taken from three independent experiments determined as described in the materials and methods. Values are means ± S.E.M; * P<0.005 and ** P<0.001 by the Mann-Whitney U test.

The fluorescence complementation signal resulting from VE-PTP-VN and VE-cadherin-VC co-expression in HEK-293T versus the VE-PTP-VN and the control pairings is shown,Fig 4A. Qualitative assessment of the images suggested that the control pairings resulted in a lower fluorescence signal. It was not possible to test reverse pairings in which the N- or C-terminal vYFP sequences were swapped as the VE-cadherin-VN construct was not successfully cloned. In order to obtain a more robust interpretation of the data, quantitative comparisons were performed between the fluorescence signal obtained with the cognate pairing and control pairings. Quantitative analysis of BiFC data has previously been performed in other studies of cytosolic proteins by calculating the ratio of BiFC fluorescent intensity relative to an expressed Cerulaen control protein to normalize for protein expression levels [29, 33]. Here we measured the BiFC fluorescent intensity of individual cells with the CellMask DeepRed membrane stain as a reference, and calculated the BiFC/DeepRed ratio. This quantitative analysis approach was validated using the well-characterized transcription factors bJun and bFos which are known to form a hetero-dimer (positive control), and bJun and the deletion mutant bΔFos which lacks the interaction domain (negative control) (S1–S4 Figs). The signal to noise value S/N = 7.7 (determined by dividing the median ratio value for the positive pairing by the median ratio value for the negative pairing) correlates closely with published values using a similar approach [29], as does the calculated fold-difference (6-fold) in mean BiFC fluorescence intensity between the positive and negative control. Application of both approaches to the VE-PTP-VN/VE-cadherin-VC pairing resulted in S/N values of 6.5 (VE-PTP-VN/VE-cadherin-VC) and 2.3 (VE-PTP-VN/control). The calculated mean BiFC values for VE-PTP-VN/VE-cadherin-VC were significantly higher (2- to 3-fold) when compared to controls indicating a specific interaction (Fig 4B). This comparison of the two approaches for quantitation indicates that both methods give similar results and in subsequent BiFC experiments data is presented as the mean BiFC value for each pairing for simplicity.

### Analysis of the VE-PTP / VE-cadherin interface

**Fig 5:**
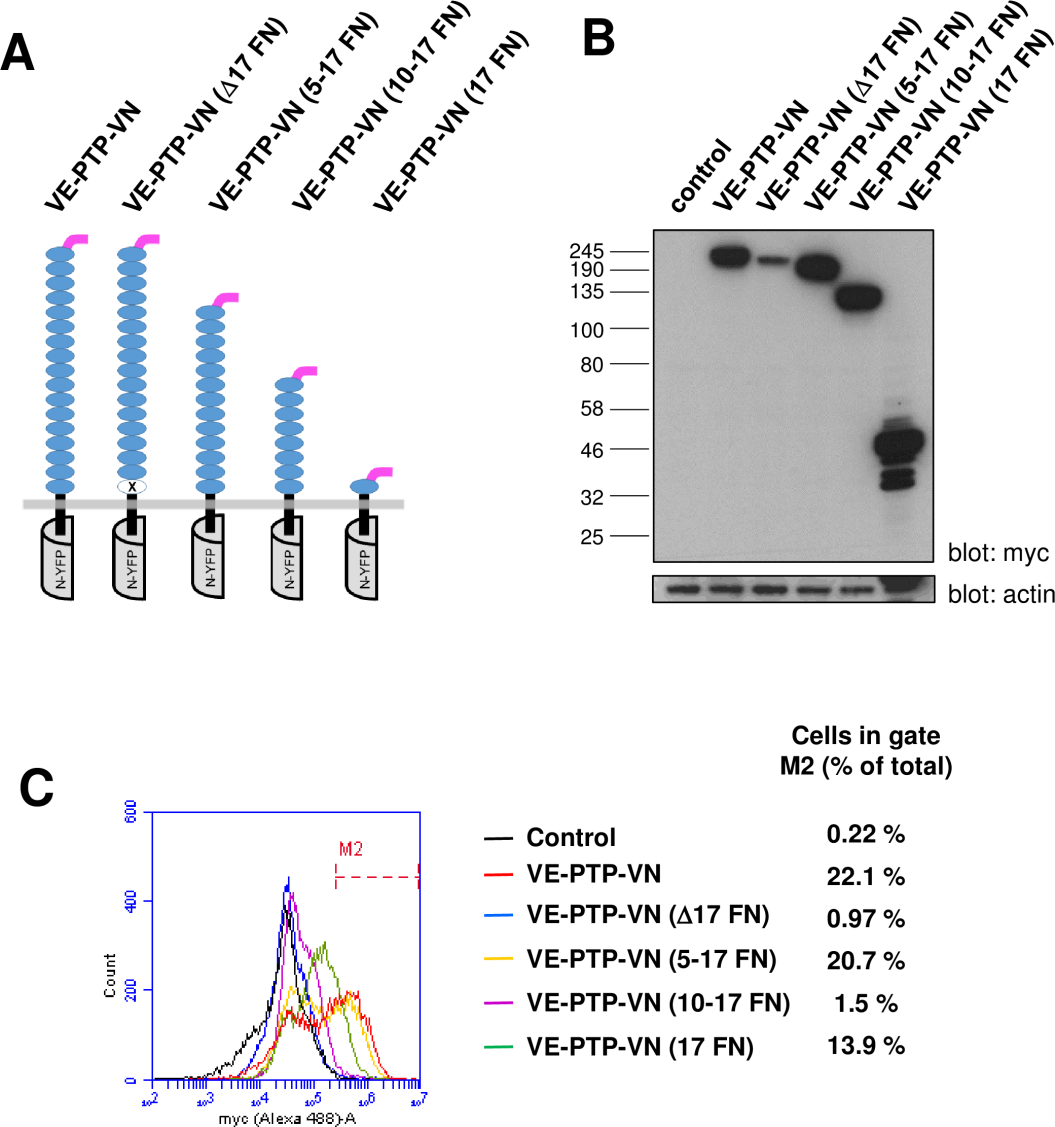
Expression analysis of VE-PTP ectodomain truncation and deletion constructs. **(A)** Representation of the VE-PTP BiFC constructs indicating the position of the deleted 17th FNIII domain and truncations; **(B)** Western blotting of cell lysates from transfected HEK-293T cells showing the relative expression of each construct with β-actin as a loading control; **(C)** Cell-surface expression analysis by flow cytometry following transfection of HEK-293T cells with the constructs indicated. Data are representative of two experiments and values indicate the percentage of cells with cell surface expression of the construct.

Previously, studies by Nawroth et al [27] used a series of VE-PTP and VE-cadherin truncation mutants in co-immunoprecipitation studies to define the region of interaction on both molecules. The results suggested that the domains proximal to the membrane (i.e. 5^th^ cadherin domain of VE-cadherin and 17^th^ FNIII-like domain of VE-PTP) are sufficient to facilitate this interaction and other parts of the molecules (ectodomains, transmembrane domains and intracellular regions) are not required [27]. Here we used both a VE-PTP deletion mutant (in which the 17^th^ FNIII-like domain has been removed) and VE-PTP constructs (in which the first 4, 9 or 16 FNIII-like domains of VE-PTP have been truncated) in live cell BiFC assays to further investigate the molecular basis of the VE-PTP with VE-cadherin interaction (Fig 5A). In analysis of whole-cell lysates by western blotting, expression of all constructs was observed close to the predicted molecular weight at a similar level to that of VE-PTP-VN, with the exception of VE-PTPΔ17FN which was expressed at substantially lower levels (Fig 5B). Expression analysis of the constructs by flow-cytometry indicated that cell-surface expression of VE-PTP-VN was detectable in 22% of cells (Fig 5C). Truncation of the FNIII-like domains 1–4 had a negligible effect on cell surface expression while further truncation of domains 1–16 caused a modest reduction (from 22% to 13.9%); however, surprisingly truncation of domains 1–9 caused an almost complete loss of cell surface expression. Possible explanations for this finding are that the region between domains 5 and 9 is important for cell surface expression, or that cell surface expression is not solely determined by the size of the ectodomain but by a combination of factors such as size, sequence and glycosylation state. Deletion of the 17^th^ FNIII-like domain (Δ17 FN) led to an almost complete loss of cell surface, although the total expression levels for this construct are also lower (Fig 5C).

**Fig 6:**
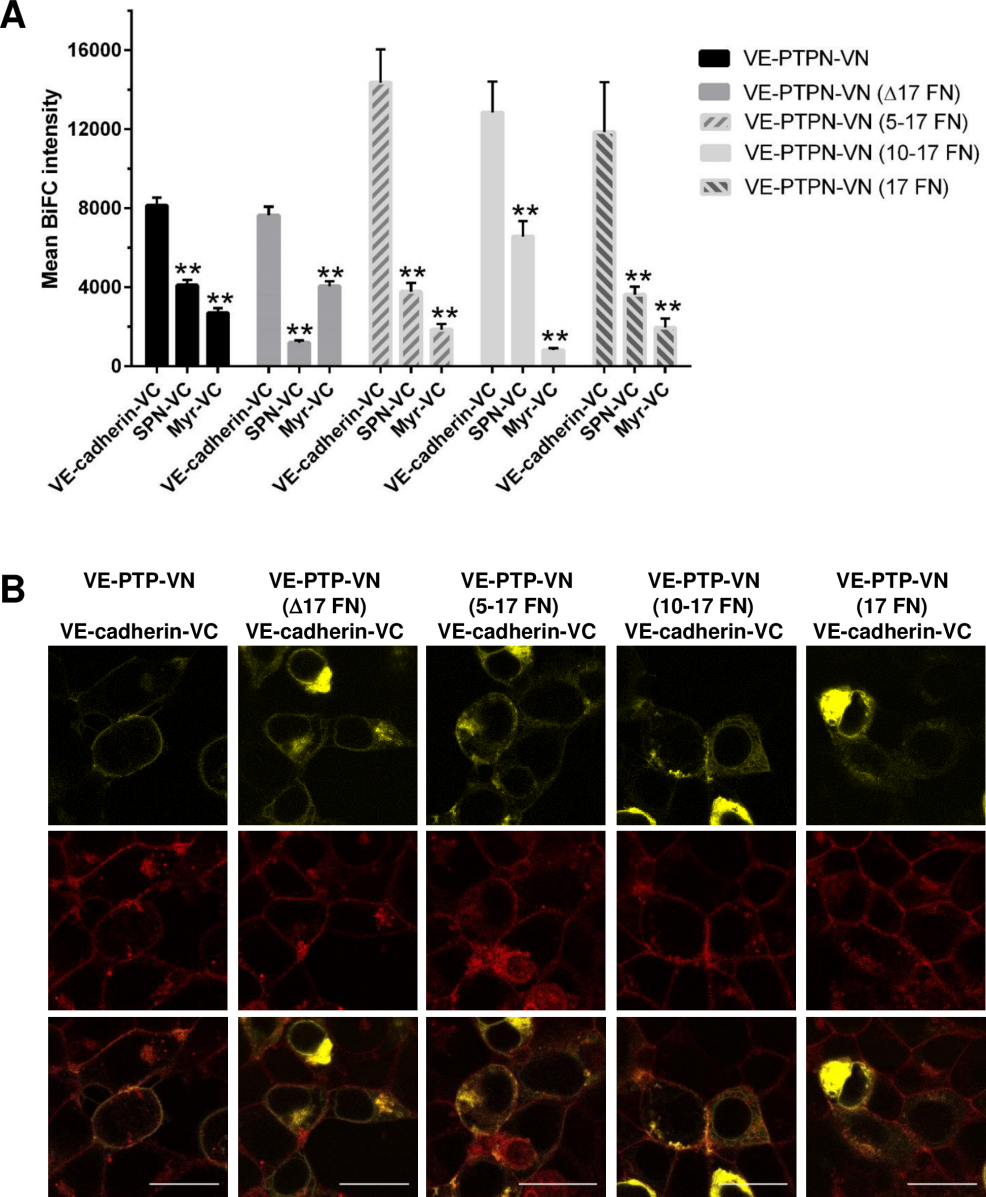
Comparison of BiFC signals from VE-cadherin co-expressed with VE-PTP and truncation or deletion constructs of VE-PTP. **(A)** Pairs of constructs were transfected into HEK-293T cells and the BiFC signal assessed. Bar graphs represent the average BiFC fluorescence intensity from all cells in three images taken from three independent experiments determined as described in the materials and methods. Values are means ± S.E.M; ** P<0.001 by the Mann-Whitney U test; **(B)** Overlay of plasma membrane staining and BiFC signal. A representative subfield at higher digital zoom showing detail at the single cell level for VE-PTP truncation and deletion mutants with VE-cadherin is shown; Scale bar 20 μm.

Comparison of average BiFC fluorescence intensity obtained with the VE-PTP-VN truncation or deletion mutants paired with VE-cadherin, relative to control proteins paired with VE-cadherin, revealed that a specific interaction was observed with all of the VE-PTP constructs (Fig 6A). A statistically significant increase in specific BiFC signal was observed with VE-PTP-VN (5–17 FN) ostensibly reflecting slightly higher expression levels of this construct relative to VE-PTP-VN. Two colour line scan analysis across the membrane using Fiji image analysis software indicated that BiFC fluorescence is coincident with plasma membrane staining on expression of either the VE-PTP-VN construct or 5–17 FN construct (S5 Fig). This finding indicates that the region consisting of FNIII-like domains 1–4 of VE-PTP is not required for interaction of this protein with VE-cadherin at the plasma membrane. However, in constructs with larger deletions domains 1–9 (10–17 FN) and 1–16 (17 FN), or where the 17^th^ domain was deleted (Δ17 FN), co-localisation of the BiFC signal and plasma membrane staining was negligible, indicating that the interaction takes place in an intracellular structure (S5 Fig). These findings are consistent with the flow cytometry data which indicated that only a negligible fraction of Δ17 FN and 10–17 FN constructs is expressed at the cell surface. They also reveal that even though the construct containing the 17^th^ domain (17 FN) reaches the cell surface, the BiFC signal is almost exclusively intracellular and is not apparent at the plasma membrane.

### Interaction of other R3-PTP family members with VE-cadherin

**Fig 7:**
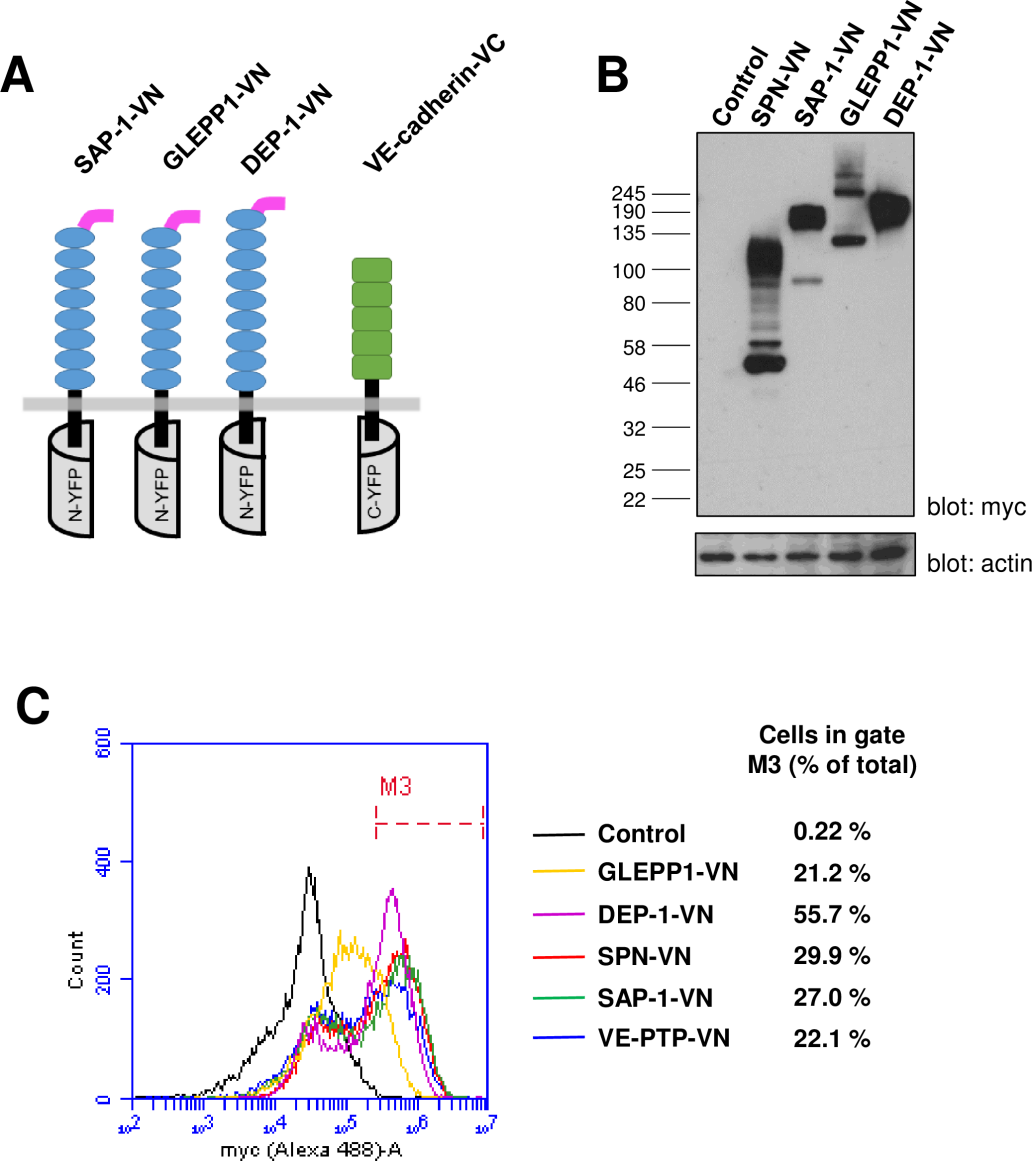
Expression analysis of other R3-PTP family members as BiFC constructs. **(A)** Representation of the BiFC constructs for DEP-1, SAP-1 and GLEPP1, have 8–9 fibronectin III-like repeats; **(B)** Western blotting of lysates from transfected HEK-293T cells using an anti-myc tag antibody with β-actin was used as a loading control; **(C)** Cell-surface expression analysis of transfected HEK-293T cells by flow cytometry using an anti-myc tag antibody and goat-anti mouse Alexa488-labelled secondary antibody. Data are representative of two experiments and values indicate the percentage of cells within gate M3 with cell surface expression of the construct.

**Fig 8:**
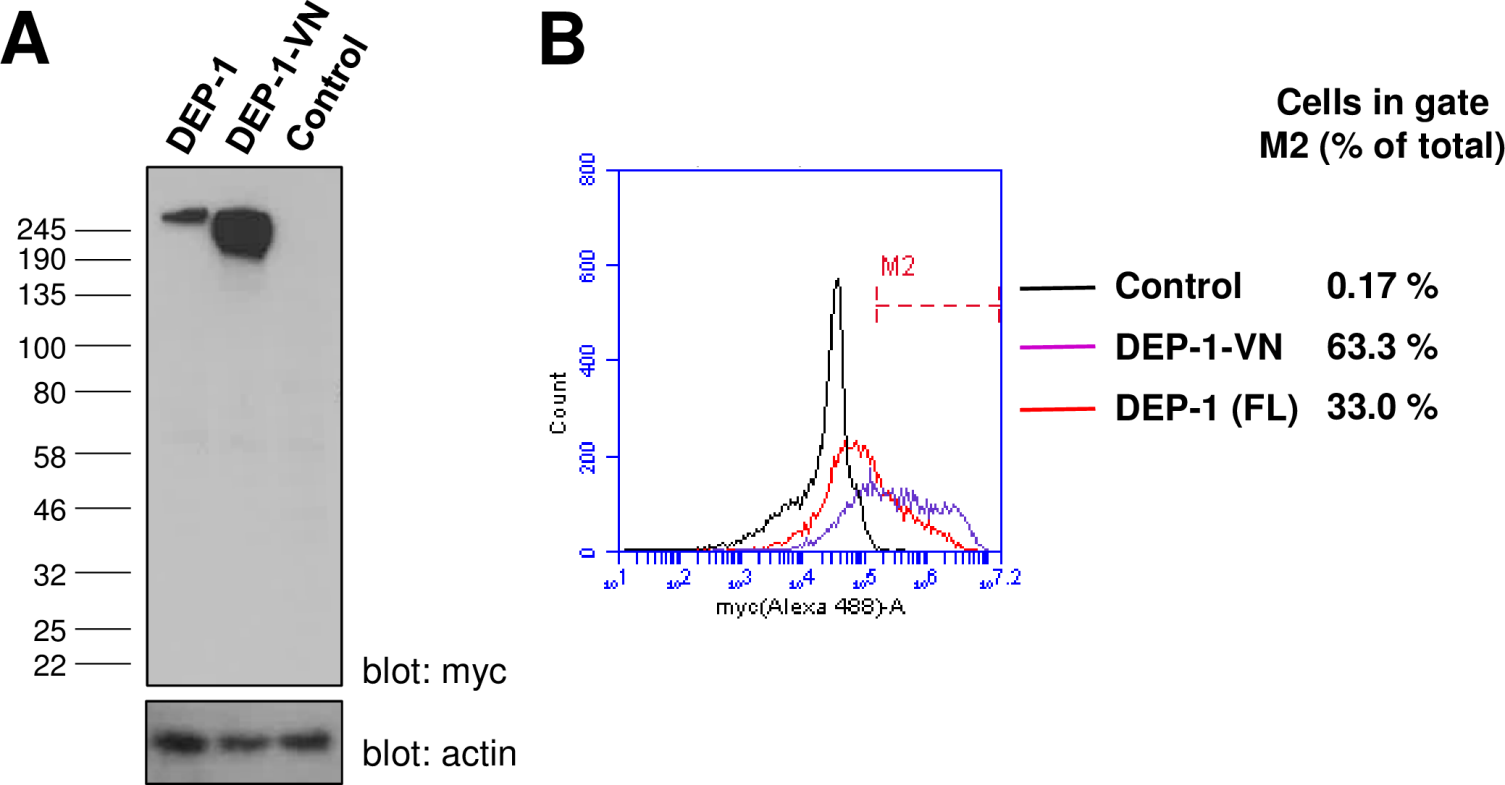
Comparison of DEP-1-VN and full-length DEP-1 (FL) expression. **(A)** Western blotting of lysates from transfected HEK-293T cells using an anti-myc tag antibody with β-actin as a loading control; **(B)** Cell-surface expression analysis by flow cytometry following transfection of HEK-293T cells. Values indicate the percentage of cells with cell surface expression of the construct.

The molecular basis for the interaction of VE-cadherin with receptor-type phosphatases was explored further by examining whether other members of the R3-PTP family (DEP-1, SAP-1 and GLEPP1) have the potential to interact with this protein. Expression analysis of whole cell lysates from transfected HEK-293T cells indicated all three constructs (Fig 7A) were expressed and displayed molecular weights significantly higher than predicted, most likely reflecting extensive glycosylation of the proteins (Fig 7B). The GLEPP1 construct was expressed at lower levels and appeared as two bands, most likely due to differential glycosylation states of the protein. Flow cytometry showed that all three constructs were expressed at the cell surface with expression detected in 22–56% of cells, which is similar to, or higher than, the percentage of cells expressing VE-PTP-VN and the control protein SPN-VN (Fig 7C). An important consideration is that truncation of intracellular sequences and generation of the BiFC fusion proteins does not adversely affect the localization of the protein. We examined this for the DEP-1-VN fusion protein by comparing it with a full-length myc-tagged DEP-1 construct. The fraction of the fusion protein, relative to total expression, that was expressed at the cell surface was similar to that of the full-length protein indicating that cell-surface localization was not significantly affected by creating the fusions (Fig 8).

**Fig 9:**
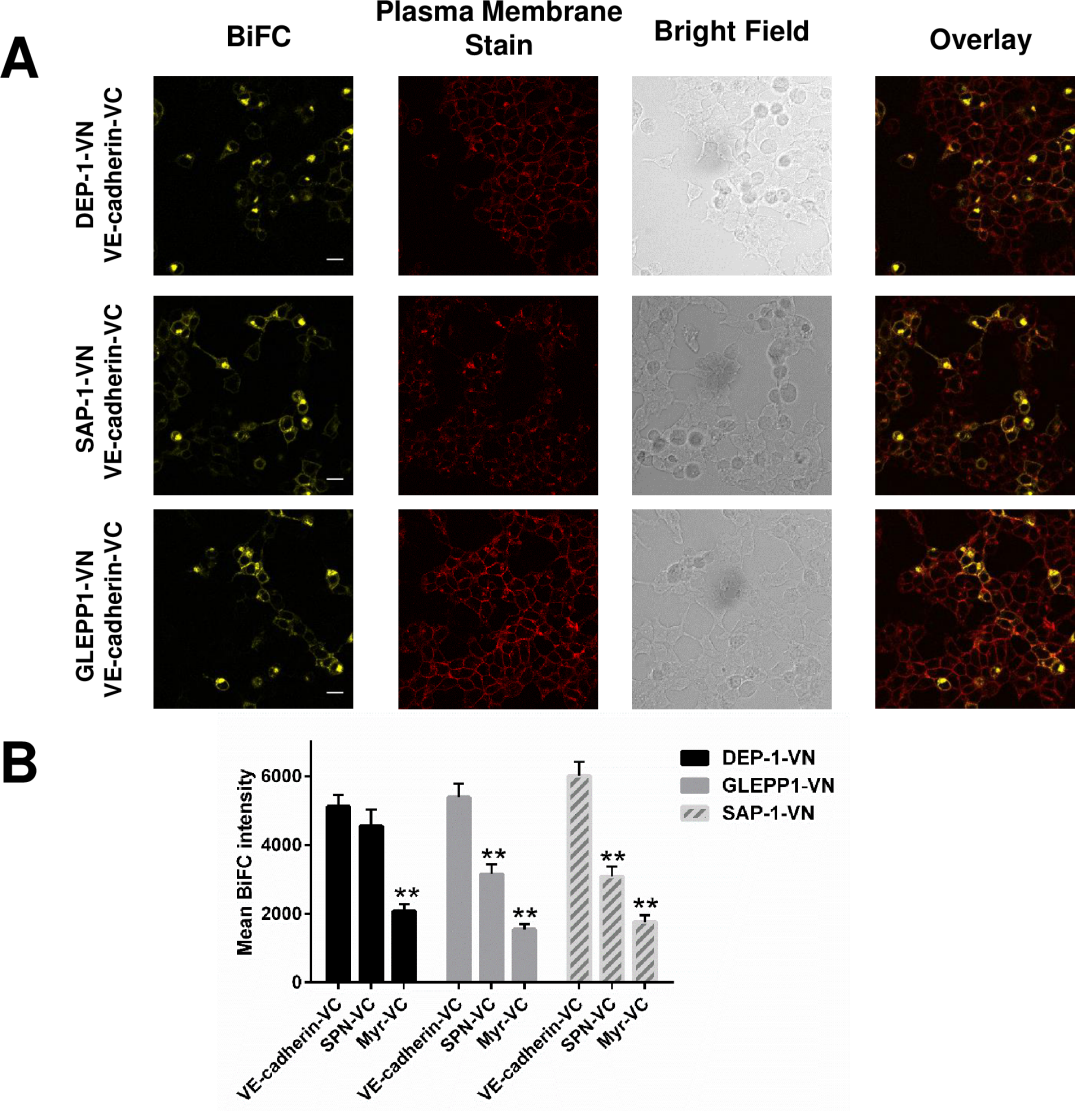
BiFC analysis of R3-PTP family members DEP-1, GLEPP1 and SAP-1 co-expressed with VE-cadherin. **(A)** The indicated pairs of constructs were transfected into HEK-293T cells and the BiFC signal assessed. Representative images are shown with plasma membrane staining, brightfield and overlayed images shown on additional panels. Data are representative of at least three independent experiments. Scale bar 20 μm. **(B)** Average BiFC fluorescence intensity from three images taken from three independent experiments. Values are means ± S.D; * P<0.001 by the Mann-Whitney U test.

Co-expression of BiFC constructs for other R3-PTP family members (DEP-1, SAP-1 and GLEPP1) with VE-cadherin resulted in a significantly higher average BiFC signal relative to the R3-PTP construct paired with myr-VC (Fig 9). The BiFC signal for GLEPP1-VN or SAP-1-VN with VE-cadherin was significantly higher than that obtained with these R3-PTPs paired with the control SPN-VC; however, interestingly in the case of the DEP-1-VN/SPN-C pairing there was no significant difference in the BiFC signal compared with DEP-1-VN/VE-cadherin-VC, suggesting that these proteins may interact specifically.

## Discussion

The molecular basis for the interaction of VE-PTP with VE-cadherin has previously been studied using co-immunoprecipitation experiments; however, it has not to-date been studied in live cells, and it is not known whether other R3 subgroup RPTPs have the potential to interact with VE-cadherin. Here we used the bimolecular fluorescence complementation technique to investigate the basis for the interaction of VE-PTP and VE-cadherin in live cells. Quantitative assessment of the fluorescence images obtained on co-expression of VE-PTP and VE-cadherin constructs containing the ectodomain and transmembrane domain of these molecules indicated a BiFC signal that is due to a specific interaction, rather than self-assembly of constructs and background fluorescence.

We had hypothesized, based on the Nawroth *et al* study [27], that deleting the 17^th^ FNIII-like domain from the ectodomain of VE-PTP would be sufficient to abolish the VE-PTP interaction with VE-cadherin; however, this was not observed in our BiFC studies. A similar BiFC signal was obtained with VE-PTP-VN, containing the full extracellular domain and transmembrane domain of VE-PTP, and VE-PTP-VN (Δ17FN), in which the 17^th^ FNII-like domain proximal to the membrane has been deleted. This was surprising since VE-PTP-VN (Δ17FN) is expressed at significantly lower levels than VE-PTP-VN. Flow cytometry showed that expressed VE-PTP-VN (Δ17FN) does not reach the cell surface and line scan analysis across the cell membrane of images indicated that the observed BiFC signal was predominantly intracellular, most likely in the endoplasmic reticulum or Golgi.

In addition to the differences in experimental approach (co-immunoprecipitation vs live cell BiFC) between our study and the Nawroth *et al* study, there are differences in the constructs used. In the construct VE-PTP-VN (Δ17FN) the 17^th^ FNIII domain of VE-PTP was deleted, which brings the 16^th^ FNIII domain proximal to the transmembrane domain, while the earlier study used a construct in which FNIII domains 1–16 had been deleted and a construct which expressed only the 17^th^ domain. Flow cytometry analysis of VE-PTP truncation constructs to determine cell surface expression was not reported in the Nawroth *et al* study, so it is possible that interactions observed in the co-immunoprecipitation reflect interaction of protein in the cytoplasm, as in our study. Our analysis of VE-PTP truncation mutants in which FNIII-like domains 1–4, 1–9 and 1–16 have been deleted demonstrated that this region is dispensable for interaction with VE-cadherin, and that a construct consisting of only the 17^th^ FNIII-like domain and transmembrane domain can still interact with VE-cadherin. This observation suggests that the 17^th^ FNIII-like domain and transmembrane domains of VE-PTP are important for the interaction with VE-cadherin and are in agreement with Nawroth *et al*; however, the interaction observed in our study was predominantly intracellular rather than at the plasma membrane.

Our measurements of the cellular BiFC levels with various VE-PTP fusion constructs co-expressed with VE-cadherin fusion constructs reflect the signal from the whole cell and do not distinguish intracellular versus plasma membrane bound signals. Attempts to accurately quantitate the two fractions by various image analysis approaches are hampered by the fact that in some, but not all cells, a substantial fraction of the BiFC signal originates from the endoplasmic reticulum and Golgi which lies in close proximity to the plasma membrane and thereby gives an over-estimate of the plasma membrane BiFC signal. Nonetheless the combination of BiFC in live cells, flow cytometry and image analysis by line scanning has allowed us to confirm that the 17^th^ FNIII-like domain of VE-PTP alone is not sufficient to support the interaction of VE-PTP and VE-cadherin at the plasma membrane.

A key question is whether the BiFC interaction observed in intracellular structures represents a physiologically relevant interaction, or is a result of the heterologous expression system used. In other BiFC membrane protein studies it has been reported that expression of a non-specific partner protein, or a mutant that fails to form a complex, leads to loss of cell surface membrane protein expression, with retention of the BiFC signal inside intracellular compartments [39, 40]. Nonetheless, while it is known that the protein in these intracellular structures, endoplasmic reticulum and Golgi may not be fully processed and folded, it is also recognised that for many membrane proteins, structural complex formation takes place in ER prior to trafficking to the pre-formed complex to the cell surface [41]. This phenomenon has been well documented for G protein-coupled receptors, where homo- or hetero-dimerization and complex formation with receptor activity-modifiying proteins (RAMPs) play a key role in trafficking of the pre-formed complex to the cell surface, and has also been reported for the growth hormone receptor and several other membrane proteins [42–45]. Further studies are required to ascertain whether the observed intracellular BiFC signal represents non-specific assembly of immature proteins, or whether it represents a pre-formed intracellular VE-PTP and VE-cadherin complex prior to trafficking to the cell membrane. In cells endogenously co-expressing both proteins it is not known whether complex formation occurs primarily in the ER, plasma membrane or both.

Analysis of the potential of other R3-PTPs (DEP-1, SAP-1 and GLEPP1) to interact with VE-cadherin revealed that the series of 8–9 extracellular FNIII-like domains and transmembrane region from any of the R3-PTPs has the potential to mediate a direct interaction with VE-cadherin. This is of particular interest with regard to DEP-1 since it is expressed in endothelial cells together with VE-cadherin, its localization at inter-endothelial cell contacts overlaps with VE-cadherin, an interaction of DEP-1 with other endothelial cell tight junction proteins has been identified, and a role in regulation of endothelial cell permeability has been documented [46–48]. We are not aware of reports of SAP-1 and GLEPP1 expression in endothelial cells, and since VE-cadherin expression is restricted to this cell type the observed interaction is unlikely to be of physiological relevance. Nonetheless the results provide insight into the molecular basis of the interaction suggesting that the interaction of VE-PTP and VE-cadherin, or other R3-PTPs with VE-cadherin, can be mediated by several different FNIII domains, and potentially their transmembrane domains. Since sequence identity among the FNIII-like domains of R3-PTP is low this finding is interesting since it suggests the molecular basis of the interaction between the VE-PTP and VE-cadherin is quite different from the interaction of fibronectin FNIII-10 with integrins, where a specific RGD (Arg-Gly-Asp) sequence on an exposed loop mediates the interaction, and in fact there is no corresponding conserved sequence in the R3-PTPs. The potential role of the transmembrane domain in mediating the interaction should not be overlooked since it has been reported that other receptor-type phosphatases form dimers via this region [49]; however, further studies are required to define the relative roles of the ectodomain and transmembrane domain in interaction of VE-PTP with VE-cadherin.

Our use of two different control constructs (SPN-VC and Myr-VC) in the BiFC experiments resulted in different average background fluorescent intensity values. In most cases use of the myr-VC control gave lower levels of BiFC intensity than the SPN-VC control underscoring the need to choose controls carefully. The observation that DEP-1 co-expressed with the SPN control was not significantly different from the DEP-1/VE-cadherin combination, but gave significantly higher levels of fluorescence than DEP-1 with the Myr-VC control could be interpreted as a *bona fide* interaction between DEP-1 and SPN although due to the variability between the controls some caution must be exercised. Although both proteins are expressed in leukocytes we are not aware of any previous reports documenting this interaction. The finding highlights the importance of using several controls in BiFC studies with membrane proteins which we believe are more prone to artefactual results than studies of freely diffusing cytoplasmic proteins, since the sub-cellular location of these proteins is restricted to the plane of the membrane. The R3-PTP BiFC constructs used in this study were truncated following the transmembrane domain, with the intracellular sequence replaced by either the N- or C-terminal sequence of vYFP. Comparison of the fraction of cell-surface expression of the DEP-1-VN construct with the full-length DEP-1 indicated that the BiFC construct exhibited a similar level of cell-surface expression as DEP-1, suggesting that the generation of the fusion protein did not detract from effective trafficking of the protein.

In summary, association of VE-PTP with VE-cadherin is a key regulator of endothelial cell-cell interaction that maintains the integrity of blood vessels. Under conditions of inflammation or immune responses, these molecules dissociate allowing leukocytes to migrate through the endothelial cell lining into the extravascular tissue. Our results, using a live cell assay, demonstrate the importance of the ectodomain and transmembrane domain of VE-PTP in the interaction with VE-cadherin, and show that DEP-1 and other R3-PTPs have the potential to interact with VE-cadherin, raising questions as to the structural basis for the interaction of these molecules.

## Supporting information

S1 Table Synthetic DNA sequences and oligonucleotides used to generate signal peptides and epitope tags in expression constructs. (DOCX)

S1 Fig Expression of Jun and Fos BiFC constructs used for validation of quantitative analysis. (DOCX)

S2 Fig Comparison of BiFC signal from co-expressed Jun / Fos versus Jun / ΔFos. (DOCX)

S3 Fig Distribution of fluorescent intensity ratios (BiFC-YFP / DeepRed) in individual cells. (DOCX)

S4 Fig Average BiFC fluorescent intensity of Jun and Fos (positive control) and Jun and ΔFos (negative control). (DOCX)

S5 Fig Analysis of sub-cellular distribution of the BiFC signal by two-colour line scan across the plasma membrane. (DOCX)

## Abbreviations

RPTPs: Receptor-type protein tyrosine phosphatases
BiFC: bimolecular fluorescence complementation
DEP-1: density-enhanced phosphatase 1
VE-PTP: vascular endothelial protein tyrosine phosphatase
SAP-1: stomach cancer-associated protein tyrosine phosphatase 1
GLEPP1: glomerular epithelial protein 1
PTPRQ: receptor-type protein tyrosine phosphatase Q
FNIII: fibronectin III
vYFP: Venus yellow fluorescent protein
SPN: sialophorin
