# Supplementary material for: Defining the molecular basis of interaction between R3 receptor-type protein tyrosine phosphatases and VE-cadherin
Source: PLoS One. 2017 Sep 19;12(9):e0184574. doi: 10.1371/journal.pone.0184574 (PMC5604967; doi:10.1371/journal.pone.0184574)
Supplement: S1 Table — (DOCX) [file pone.0184574.s001.docx]

**S1 Table.** Synthetic DNA sequences and oligonucleotides used to generate signal peptides and epitope tags in expression constructs.

| **Protein name and epitope tag** | **DNA Sequence (5’->3’)** | **Restriction sites** |
| --- | --- | --- |
| VE-PTP-HA | GGGCCCACCATGCTGAGCCATGGAGCCGGGTTGGCCTTGTGGATCACACTGAGCCTGCTGCAGACTGGACTGGCGGAGCCAGAGTACCCATACGATGTTCCAGATTACGCTTCTCGAG | *Apa*I *- Xho*I |
| DEP-1-HA | GGGCCCACCATGAAGCCGGCGGCGCGGGAGGCGCGGCTGCCTCCGCGCTCGCCCGGGCTGCGCTGGGCGCTGCCGCTGCTGCTGCTGCTGCTGCGCCTGGGCCAGATCCTGTGCGCAGGTGGCTACCCATACGATGTTCCAGATTACGCTTCTCGAG | *Apa*I *- Xho*I |
| GLEPP1-HA | GGGCCCACCATGGGGCACCTGCCCACGGGGATACACGGCGCCCGCCGCCTCCTGCCTCTGCTCTGGCTCTTTGTGCTGTTCAAGAATGCTACAGCTTTCCATGTATACCCATACGATGTTCCAGATTACGCTTCTCGAG | *Apa*I *- Xho*I |
| SAP-1-HA | GGGCCCACCATGGCTGGGGCTGGCGGGGGCCTCGGGGTCTGGGGGAACCTGGTGCTGCTGGGCCTGTGCAGCTGGACAGGGGCCAGGGCGCCTGCCCCCTACCCATACGATGTTCCAGATTACGCTTCTCGAG | *Apa*I *- Xho*I |
| SPN-HA | GGGCCCACCATGGCCACGCTTCTCCTTCTCCTTGGGGTGCTGGTGGTAAGCCCAGACGCTCTGGGGAGCACAACATACCCATACGATGTTCCAGATTACGCTCGAATTC | *Apa*I *- Eco*RI |
|  |  |  |
| VE-PTP-myc* | (PTPRB-A) gatcgggcccaccatgctgagccatggagcc  (PTPRB-B) ctacctcgagacaggtcctcctctgagatcagcttctgctc tggctccgccagtcc | *Apa*I *- Xho*I |
| DEP-1-myc | GGGCCCACCATGAAGCCGGCGGCGCGGGAGGCGCGGCTGCCTCCGCGCTCGCCCGGGCTGCGCTGGGCGCTGCCGCTGCTGCTGCTGCTGCTGCGCCTGGGCCAGATCCTGTGCGCAGGTGGCGAGCAGAAGCTGATCTCAGAGGAGGACCTGTCTCGAG | *Apa*I *- Xho*I |
| GLEPP1-myc | GGGCCCACCATGGGGCACCTGCCCACGGGGATACACGGCGCCCGCCGCCTCCTGCCTCTGCTCTGGCTCTTTGTGCTGTTCAAGAATGCTACAGCTTTCCATGTAGAGCAGAAGCTGATCTCAGAGGAGGACCTGTCTCGAG | *Apa*I *- Xho*I |
| SAP-1-myc | GGGCCCACCATGGCTGGGGCTGGCGGGGGCCTCGGGGTCTGGGGGAACCTGGTGCTGCTGGGCCTGTGCAGCTGGACAGGGGCCAGGGCGCCTGCCCCCGAGCAGAAGCTGATCTCAGAGGAGGACCTGTCTCGAG | *Apa*I *- Xho*I |
| SPN-myc | GGGCCCACCATGGCCACGCTTCTCCTTCTCCTTGGGGTGCTGGTGGTAAGCCCAGACGCTCTGGGGAGCACAACAGAGCAGAAGCTGATCTCAGAGGAGGACCTGCGAATTC | *Apa*I *- Eco*RI |
|  |  |  |

* The VE-PTP-myc sequence was generated by PCR using the primers indicated
