# Supplementary material for: Defining the molecular basis of interaction between R3 receptor-type protein tyrosine phosphatases and VE-cadherin
Source: PLoS One. 2017 Sep 19;12(9):e0184574. doi: 10.1371/journal.pone.0184574 (PMC5604967; doi:10.1371/journal.pone.0184574)
Supplement: S1 Fig — (DOCX) [file pone.0184574.s002.docx]

## S1 Figure. Expression of Jun and Fos BiFC constructs used for validation of quantitative analysis


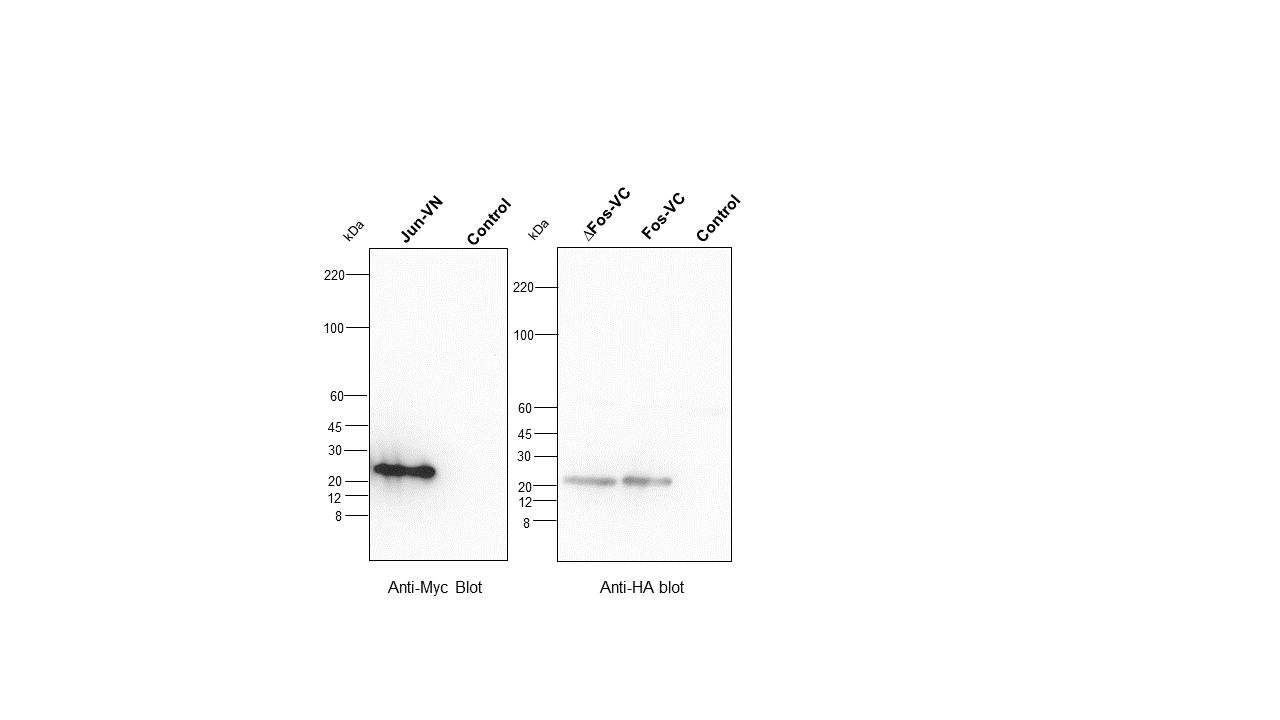


Western blotting of cell lysates from transfected HEK-293T cells with either anti-myc or anti-HA antibodies confirmed the expression of the constructs at the expected molecular weight. The constructs were obtained from Addgene: pBiFC-bJunVN155(I152L) (Plasmid #27098); pBiFC-bFosVC155 (Plasmid #22013); pBiFC-bFOSDeltaZipVC155 (Plasmid #22014)
