# Supplementary material for: Defining the molecular basis of interaction between R3 receptor-type protein tyrosine phosphatases and VE-cadherin
Source: PLoS One. 2017 Sep 19;12(9):e0184574. doi: 10.1371/journal.pone.0184574 (PMC5604967; doi:10.1371/journal.pone.0184574)
Supplement: S2 Fig — (DOCX) [file pone.0184574.s003.docx]

## S2 Figure. Comparison of BiFC signal from co-expressed Jun / Fos versus Jun / ΔFos


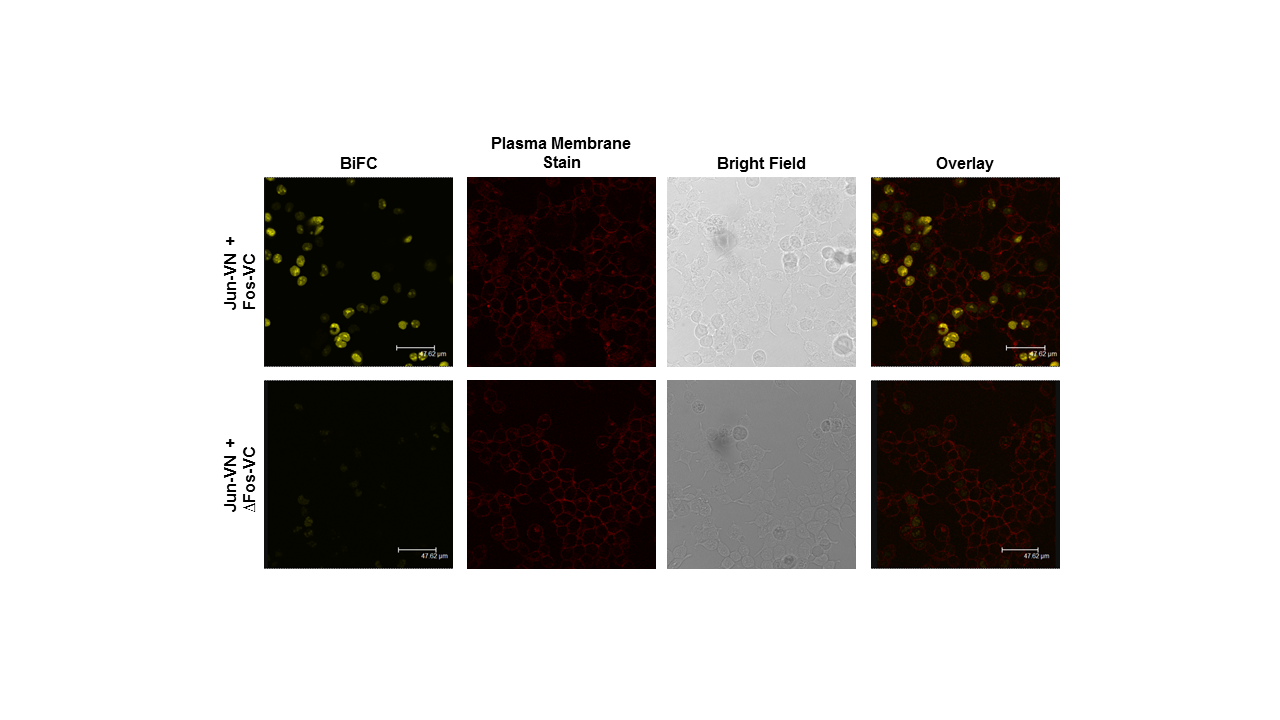


Constructs expressing Jun and Fos (positive control) and Jun and ΔFos (negative control) as fusions with fragments of Venus YFP were transfected into HEK-293T cells. The constructs complement each other to yield a BiFC signal. Plasma membrane staining (CellMask DeepRed), brightfield and overlayed images are shown on additional panels. The scale bar is 48 μm and data are representative of at least three independent experiments
