# Supplementary material for: Defining the molecular basis of interaction between R3 receptor-type protein tyrosine phosphatases and VE-cadherin
Source: PLoS One. 2017 Sep 19;12(9):e0184574. doi: 10.1371/journal.pone.0184574 (PMC5604967; doi:10.1371/journal.pone.0184574)
Supplement: S3 Fig — (DOCX) [file pone.0184574.s004.docx]

## S3 Figure. Distribution of fluorescent intensity ratios (BiFC-YFP / DeepRed) in individual cells


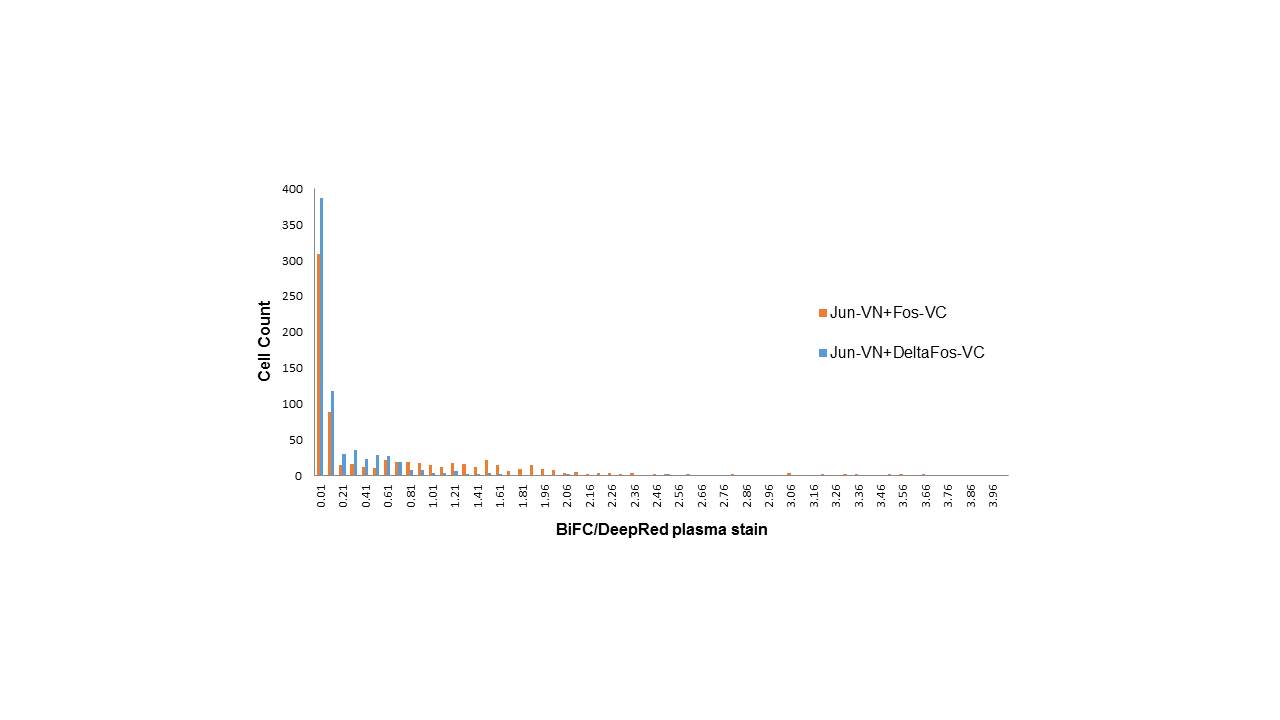


For images from the Jun and Fos (positive control, orange) and Jun and ΔFos (negative control, blue) ImageJ software was used to trace the border of each cell and determine the average pixel intensity in both the yellow and red channels of the selected region, with background subtracted. Ratios from individual cells are plotted in the histogram. Protein interaction results in higher fluorescence ratios in all or a subset of cells while when no interaction (*i.e.* only self-assembly) is observed most cells have a low fluorescence ratio. The signal to noise (S/N) was obtained by dividing the median ratio value for the positive pairing by the media ratio value for the negative pairing. A S/N ratio = 7.7 was obtained which agrees with previous results obtained by Kodama and Hu (2010) using a related approach. Images were from three independent experiments.
