# Supplementary material for: Defining the molecular basis of interaction between R3 receptor-type protein tyrosine phosphatases and VE-cadherin
Source: PLoS One. 2017 Sep 19;12(9):e0184574. doi: 10.1371/journal.pone.0184574 (PMC5604967; doi:10.1371/journal.pone.0184574)
Supplement: S4 Fig — (DOCX) [file pone.0184574.s005.docx]

## S4 Figure. Average BiFC fluorescent intensity of Jun and Fos (positive control) and Jun and ΔFos (negative control)


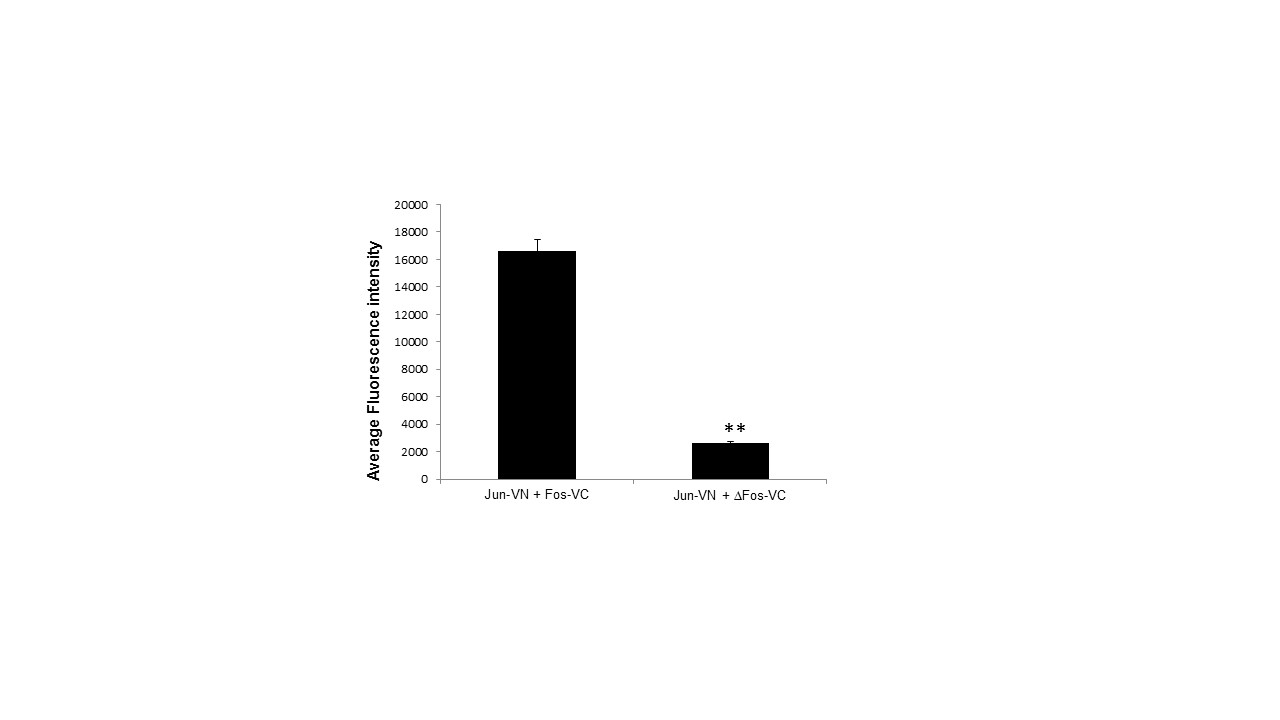


The Jun and Fos (positive control) results in a higher average fluorescent intensity than the Jun and ΔFos (negative control). A 6-fold difference in the mean BiFC fluorescence intensity was observed. Fluorescence intensity from three images taken from three independent experiments was quantitated as described in the materials and methods. Values are means ± S.D; ** P<0.001 by the Mann-Whitney U test.
