# Supplementary material for: Defining the molecular basis of interaction between R3 receptor-type protein tyrosine phosphatases and VE-cadherin
Source: PLoS One. 2017 Sep 19;12(9):e0184574. doi: 10.1371/journal.pone.0184574 (PMC5604967; doi:10.1371/journal.pone.0184574)
Supplement: S5 Fig — (DOCX) [file pone.0184574.s006.docx]

## S5 Figure. Analysis of sub-cellular distribution of the BiFC signal by two-colour line scan across the plasma membrane

**A. Co-expression of VE-PTP-VN and VE-cadherin-VC**

**
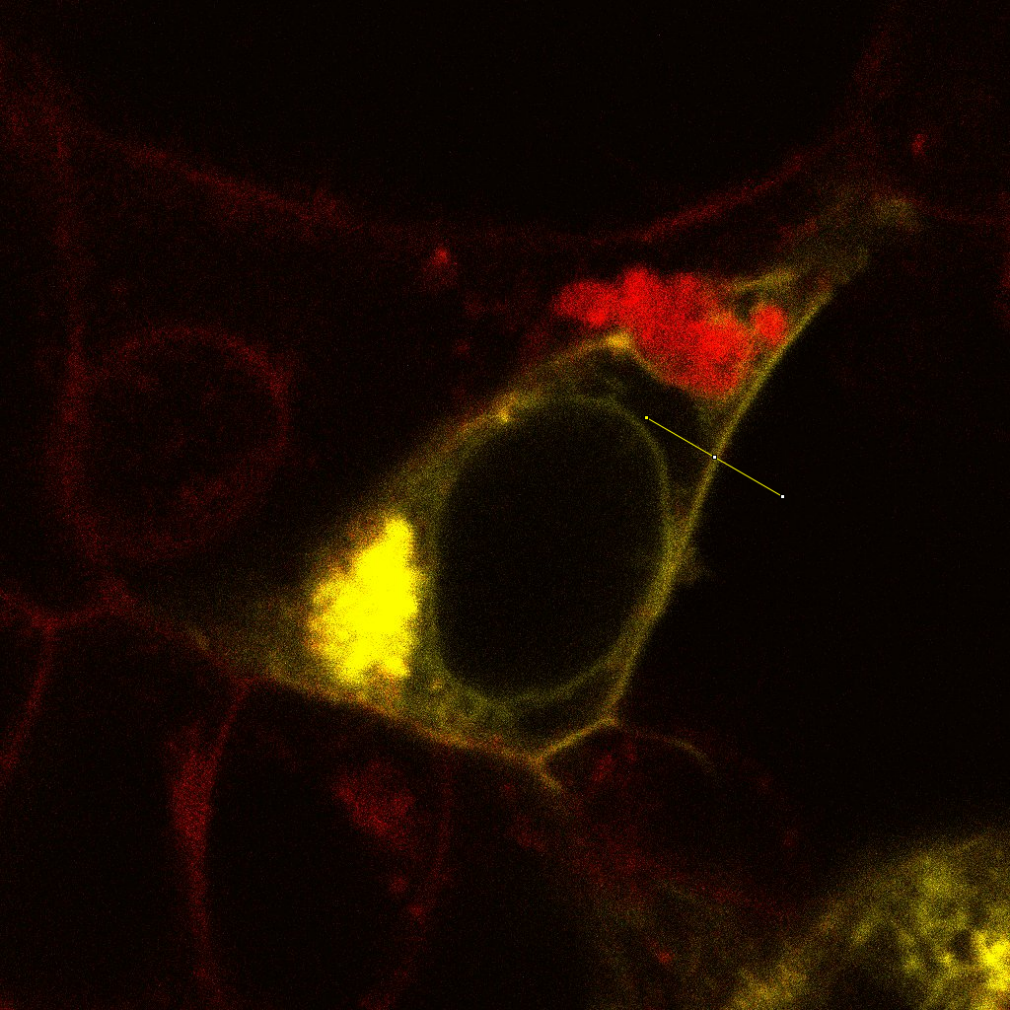
**

**B. Co-expression of VE-PTP-VN (Δ17 FN) and VE-cadherin-VC**

**
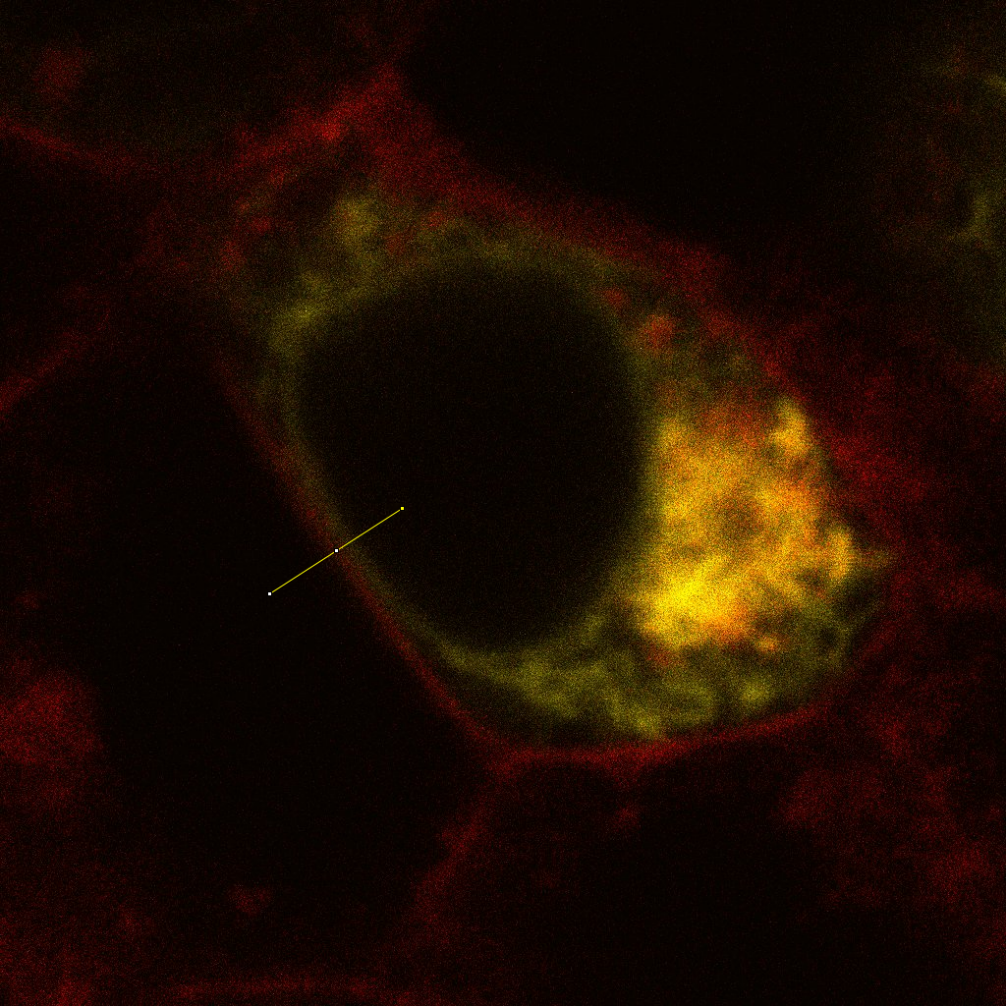
**

**C. Co-expression of VE-PTP-VN (5-17 FN) and VE-cadherin-VC**

**
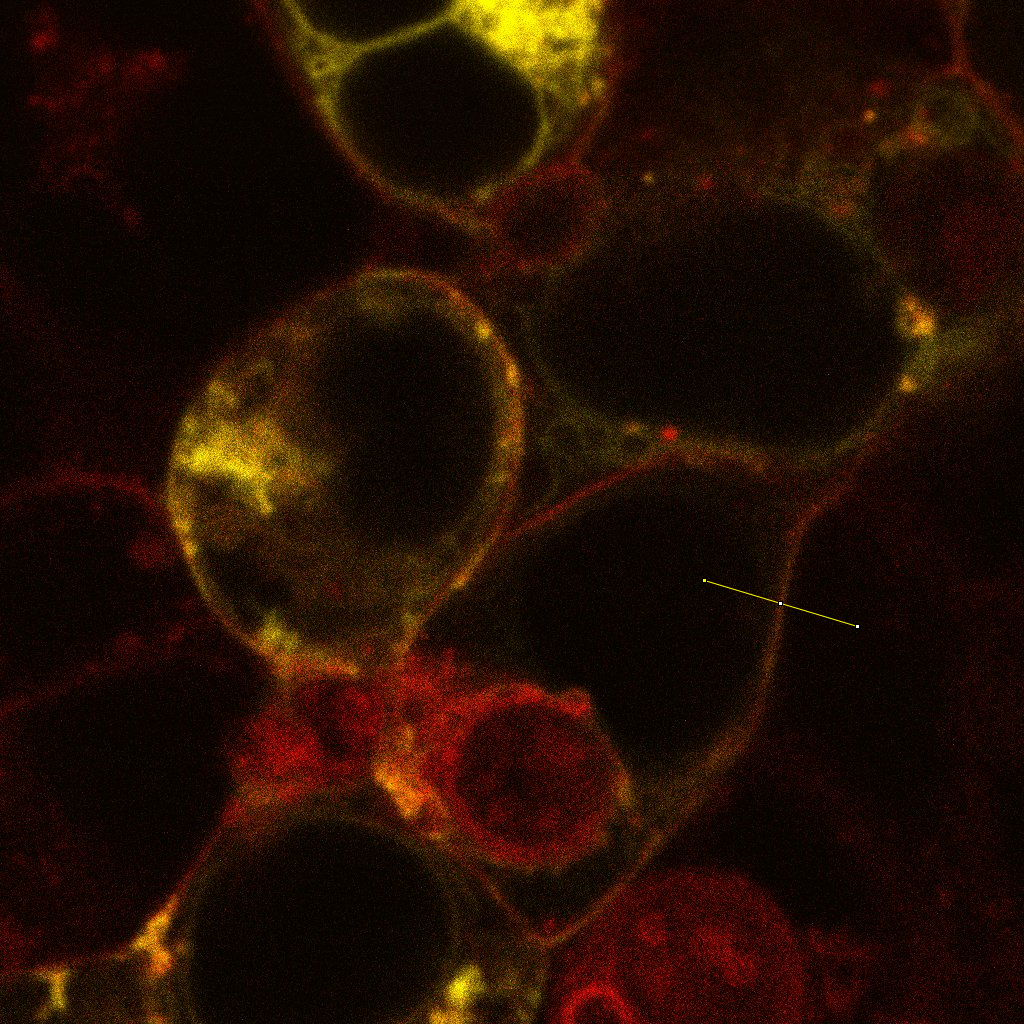
**

**D. Co-expression of VE-PTP-VN (10-17 FN) and VE-cadherin-VC**

**
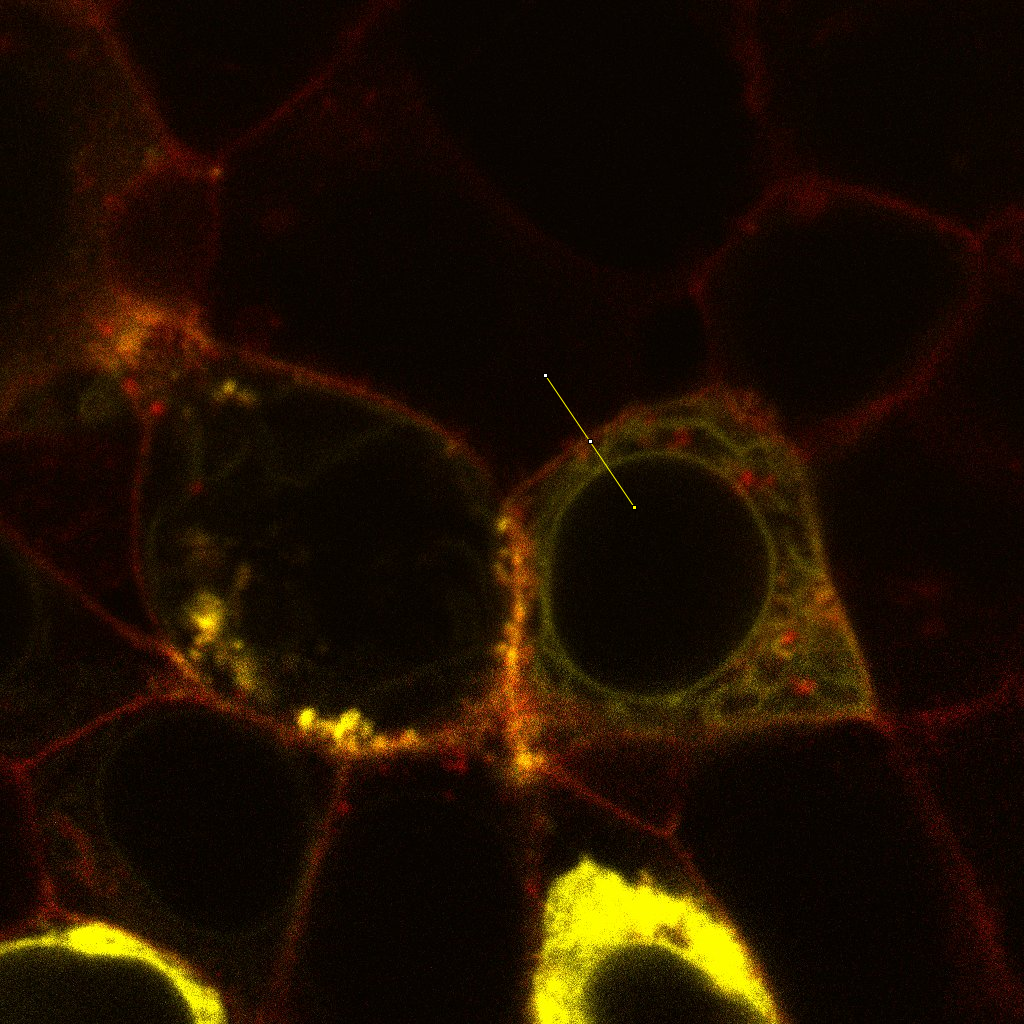
**

**E. Co-expression of VE-PTP-VN (17 FN) and VE-cadherin-VC**

**
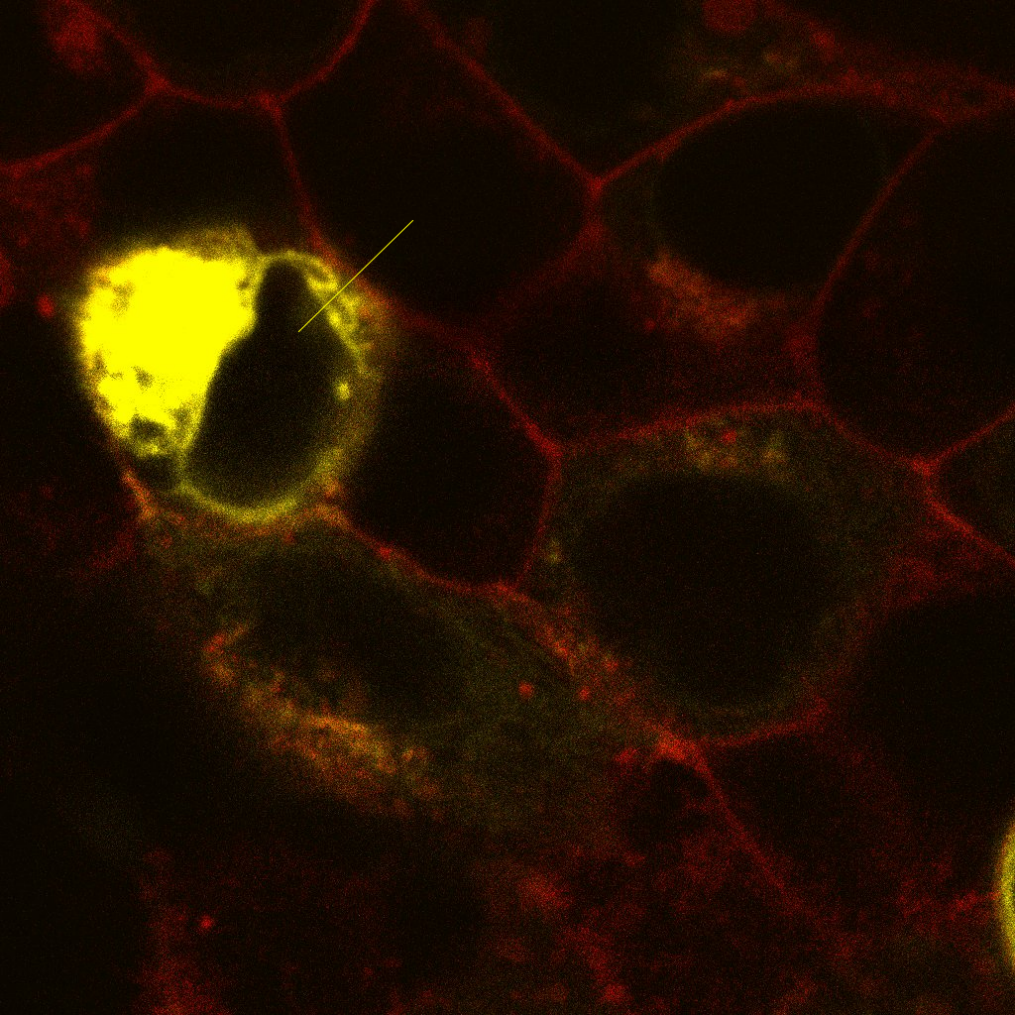
**

HEK-293T cells were transfected with constructs consisting of the ectodomain and transmembrane domains of VE-PTP and VE-cadherin as fusions with fragments of Venus YFP to yield a BiFC signal. The plasma membrane was stained with CellMask™ Deep Red plasma membrane stain. Using a representative confocal image the yellow and red channels were super-imposed (as shown). Fluorescence intensity, determined by Fiji image analysis software, at pixels along the line (see image) from inside to the outside of the cell has been plotted. The mid-point of the line was positioned in the middle of the plasma membrane and is indicated (red triangle).
